# 2,3-Diaminopropanols Obtained from d-Serine as Intermediates in the Synthesis of Protected 2,3-l-Diaminopropanoic Acid (l-Dap) Methyl Esters

**DOI:** 10.3390/molecules25061313

**Published:** 2020-03-13

**Authors:** Andrea Temperini, Donatella Aiello, Fabio Mazzotti, Constantinos M. Athanassopoulos, Pierantonio De Luca, Carlo Siciliano

**Affiliations:** 1 Dipartimento di Scienze Farmaceutiche, Università di Perugia, Via del Liceo 1, 06123 Perugia, Italy; andrea.temperini@unipg.it; 2 Dipartimento di Chimica e Tecnologie Chimiche (CTC), Via Ponte P. Bucci, Cubo 12D, Università della Calabria, I-87036 Arcavacata di Rende (CS), Italy; donatella.aiello@unical.it (D.A.); fabio.mazzotti@unical.it (F.M.); 3 Department of Chemistry, University of Patras, GR-26504 Patras, Greece; kath@chemistry.upatras.gr; 4 Dipartimento di Ingegneria Meccanica, Energetica e Gestionale, Università della Calabria, I-87036 Arcavacata di Rende (CS), Italy; pierantonio.deluca@unical.it; 5 Dipartimento di Farmacia e Scienze della Salute e della Nutrizione, Edificio Polifunzionale, Università della Calabria, I-87036 Arcavacata di Rende (CS), Italy

**Keywords:** 2,3-l-diaminopropanoic acid, l-Dap, basic amino acids, serine, reductive amination, orthogonal protecting groups, 1,3,5-trichloro-2,4,6-triazinetrione, trichloroisocyanuric acid (TCCA), (2,2,6,6-tetramethylpiperidi-1-yl)oxyl (TEMPO), *N*-methylation

## Abstract

A synthetic strategy for the preparation of two orthogonally protected methyl esters of the non-proteinogenic amino acid 2,3-l-diaminopropanoic acid (l-Dap) was developed. In these structures, the base-labile protecting group 9-fluorenylmethyloxycarbonyl (Fmoc) was paired to the *p*-toluensulfonyl (tosyl, Ts) or acid-labile *tert*-butyloxycarbonyl (Boc) moieties. The synthetic approach to protected l-Dap methyl esters uses appropriately masked 2,3-diaminopropanols, which are obtained via reductive amination of an aldehyde prepared from the commercial amino acid *N*^α^-Fmoc-*O*-*tert*-butyl-d-serine, used as the starting material. Reductive amination is carried out with primary amines and sulfonamides, and the process is assisted by the Lewis acid Ti(O^i^Pr)_4_. The required carboxyl group is installed by oxidizing the alcoholic function of 2,3-diaminopropanols bearing the tosyl or benzyl protecting group on the 3-NH_2_ site. The procedure can easily be applied using the crude product obtained after each step, minimizing the need for chromatographic purifications. Chirality of the carbon atom of the starting d-serine template is preserved throughout all synthetic steps.

## 1. Introduction

### 1.1. The Role of l-Dap in Natural and Synthetic Compounds

2,3-l-Diaminopropanoic acid (l-Dap) is a non-proteinogenic amino acid produced by numerous plants and bacteria [1]. It is the main structural motif of albizziine, a natural non-proteinaceous free amino acid which is ubiquitously present in higher plants and in seeds [2]. The known preparative methods for the total synthesis of albizziine employs protected derivatives of l-Dap as the starting materials [3]. Bleomycins (BLMs) are structurally related glycopeptide-derived antitumor antibiotics, and they display a l-Dap amide common scaffold as the chain *N*-terminus [4,5,6,7,8]. l-Dap is also one of the most frequently found amino acids inserted in bioactive peptides, such as cyclotheonamide B, phleomycin, paenibacterin derivatives, lavendomycin, gastrin-releasing peptide receptor-specific scaffolds, capreomycin, various peptide-based antibacterials, and peptidomimetics, such as vitronectin [9,10,11,12,13,14,15,16]. Protected l-Dap derivatives were proposed as powerful inhibitors of the formation of advanced glycosylation end-products (AGEs), which are implicated in the development of diabetes mellitus and neurodegenerative Alzheimer’s and Parkinson’s diseases [17]. Advanced lipid peroxidation end-product (ALE) formation can also be modulated by the same l-Dap derivatives used to control AGE cascades, against the endogenous peroxidation of polyunsaturated fatty acids [17]. Matrix metalloproteases, containing l-Dap as the terminal carboxylate amino acid unit, were synthesized and biologically tested for their roles in the degradation and remodeling of connective tissues [18]. l-Dap is contained in staphyloferrin B, as well as in a series of synthetic and natural siderophores [19,20]. Appropriately protected l-Dap subunits were used as building blocks in the solid-phase synthesis of chelating peptide conjugates having therapeutic and diagnostic applications in tumor diseases [21]. Dismutation of superoxide species can be controlled by metal complexes of l-Dap branched peptides with divalent cations [22,23]. l-Dap dipeptides were used as reference structures to investigate the ionizability of side-chain primary amino functions in amino acids [24], and the substitution of aspartic units by l-Dap was adopted in the synthetic preparation of glycoproteins [25]. l-Dap acts as crosslinker in graphene sponges of clinical importance for hemostasis control [26], and it is a component of biosynthetic model compounds employed in studies aiming to define acyl-enzyme activities and foldings [27,28]. l-Dap-based peptide dendrimers catalyze asymmetric processes in organic synthesis [29,30]. Furthermore, l-Dap-enriched peptide backbones were obtained for gene silencing scopes [31], and synthetic orthogonally *N*^α^,*N*^β^-diprotected l-Dap-based nucleoamino acids were investigated for the assembly of cationic nucleopeptides in biomedical applications [32]. Recently, the identification of l-Dap in the Murchison chondritic meteorite [33] suggested the possible role of this non-proteinogenic amino acid as a selective molecular target to investigate prebiotic polycondensation processes of importance in establishing the appearance of life on Earth [34].

### 1.2. Synthetic Routes to l-Dap

The last decade was characterized by the disclosure of a wide range of synthetic routes to l-Dap and its analogues. Much effort was spent searching for convenient methods which could employ simple molecular scaffolds [35]. Natural amino acids were proposed as convenient starting materials; however, the use of serine was seldom investigated [36,37,38,39]. A combination of the classical series of acid- and base-labile masking groups used in the solution and solid-phase peptide synthesis was generally found advantageous. The installation of two or more amino functions in natural carbon backbones and their heterocycle analogues might present additional difficulty [40]. Another critical step is the preservation of the absolute configuration of chiral carbon atoms when molecules from the natural chiral pool might be used as enantiomerically pure starting materials. Differently *N*^α^,*N*^β^-diprotected l-Dap, as well as its mono- and di-alkylated derivatives, was obtained through a plethora of synthetic approaches [41,42,43,44,45,46,47,48,49,50,51,52]. In general, the published methods are characterized by variable overall yields in l-Dap, and they are notable for needing several steps of chromatography and for the problematic stereochemical control of reactions.

## 2. Scope of the Work

The scope of the present work was an investigation of a preparative route to appropriately protected l-Dap methyl esters, which may further be employed in branching, alkylation, cyclization, cross-linking, and solid-phase peptide synthesis. More specifically, we were attracted by the design of an advantageous synthetic procedure that might include (i) the easy replacement of protections placed on the amino functions of l-Dap, (ii) the methylation of the carboxyl function to obtain l-Dap methyl esters, and (iii) the possible use of appropriate masking groups which could enable a chemoselective *N*^β^-methylation, in order to prepare suitable scaffolds for the elaboration of alkylated l-Dap derivatives.

In particular, our synthetic plan was chalked out starting from optically pure protected d-serine. The key synthetic step of the procedure was the Ti(O^i^Pr)_4_-assisted reductive amination [53,54,55] of a protected d-serine aldehyde derivative to give a series of protected 2,3-diaminopropanols, which were oxidized under mild conditions [56,57]. It is worth noting that d-serine was used to obtain the correct l-Dap stereochemistry.

## 3. Results and Discussion

### 3.1. Retrosynthetic Analysis

**Scheme 1:**
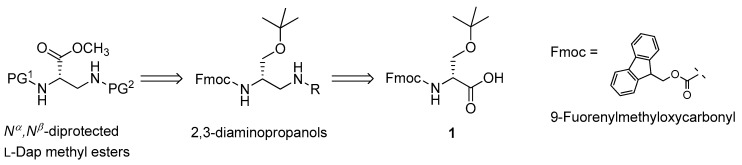
The retrosynthetic analysis for the preparation of orthogonally *N*^α^,*N*^β^-diprotected 2,3-l-diaminopropanoic acid (l-Dap) methyl esters (PG^1^ and PG^2^ are orthogonal groups).

Scheme 1 shows the retrosynthetic analysis for obtaining *N*^α^,*N*^β^-diprotected l-Dap methyl esters. Enantiomerically pure Fmoc-*O*-*tert*-butyl-d-serine (**1**), commercially available at low cost, was selected as the ideal precursor of the targeted compounds.

It was thought feasible to realize the desired 2,3-diamino framework via the synthesis of suitable intermediates having a 2,3-diaminopropanol scaffold, prepared via reductive amination of a d-serine aldehyde derivative with different primary amines and arylsulfonamides, bearing protecting groups compatible with the conditions of the solution and solid-phase peptide chemistry. The mild oxidation of alcohol group of the amine-protected 2,3-diaminopropanols could generate the carboxylic group required to complete the 2,3-diamino acid backbone. Finally, the carboxyl function can easily be methylated to afford the desired fully protected l-Dap methyl esters. The plan was designed in order to protect the 2-NH_2_ group of the methyl esters with the base-labile Fmoc group (PG^1^). In that structure, the 3-NH_2_ amino function could orthogonally be protected to give a tosyl derivative, or one with an acid-labile Boc group (PG^2^). In the 2,3-diamminopropanol intermediates, the simultaneous presence of a benzyl moiety and an orthogonal Fmoc protection on the 3- and 2-NH_2_ functions, respectively, could also support the facile interconversion of the pair of amino protections, enabling the installation of Boc (PG^1^) and Fmoc (PG^2^) groups at the 2- and 3-positions of the final l-Dap methyl ester structures.

### 3.2. Synthesis of the 2,3-Diaminopropanols

**Scheme 2:**
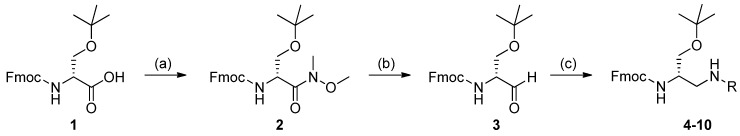
Synthesis of Fmoc-2,3-diaminopropanols **4**–**10**. Experimental conditions: preparation of the Weinreb–Nahm amide **2**, (a) 1-hydroxybenzotriazole (HOBt) monohydrate/*N*,*N*′-diisopropylcarbodiimide (DIC)/1-(3-dimethylaminopropyl)-3-ethylcarbodiimide hydrochloride (EDC), 2 h, room temperature (r.t.); then, *N*,*O*-dimethylhydroxylamine hydrochloride/DIEA/DCM, overnight, r.t. (94% yield); preparation of the Garner-like aldehyde **3**, (b) LiAlH_4_/THF, 12 min, r.t. (92% yield); reductive amination, (c) amine or arylsulfonamide/Ti(O^i^Pr)_4_/EtOH, 10 min, r.t., then NaBH_3_CN, overnight, r.t. (82%–92%, Table 1).

Scheme 2 displays our synthetic route to the Fmoc-protected 2,3-diaminopropanol intermediates **4**–**10**. These compounds were obtained via reductive amination of **3** with amines and sulfonamides. The protected amino acid **1** reacted with *N*,*O*-dimethylhydroxylamine hydrochloride [58] to give the Weinreb–Nahm amide **2**, which was recovered in a 94% yield, without need for chromatographic purification. Reduction of **2** by LiAlH_4_ afforded the corresponding α-amino aldehyde **3**, which was obtained in a 92% yield, avoiding chromatography. The structures of compounds **2** and **3** were confirmed by ^1^H- and ^13^C-NMR spectroscopy. Compound **3** was then subjected to the reductive amination with a series of primary amines and sulfonamides having different structural characteristics. Benzylamine was chosen as an ideal model to check the reaction performance. The process was assisted by the Lewis acid Ti(O^i^Pr)_4_, in the presence of sodium cyanoborohydride, a reducing agent which is widely employed in metal-assisted reactions of amino acids and their derivatives [59,60]. Primary amines result in the removal of the base-labile *N*-masking Fmoc group; however, the use of a Lewis acid prevents this practical inconvenient [61]. Compound **3** reacted smoothly with benzylamine, in the presence of the titanium(IV) species and the hydride donor, to afford **4** in excellent yields (92%), after chromatography. Thin-layer chromatography (TLC) analysis, as well as the ^1^H-NMR spectrum of the crude reaction material, highlighted the absence of unwanted by-products generated through the removal of the base-labile Fmoc protecting group.

**Figure 1:**
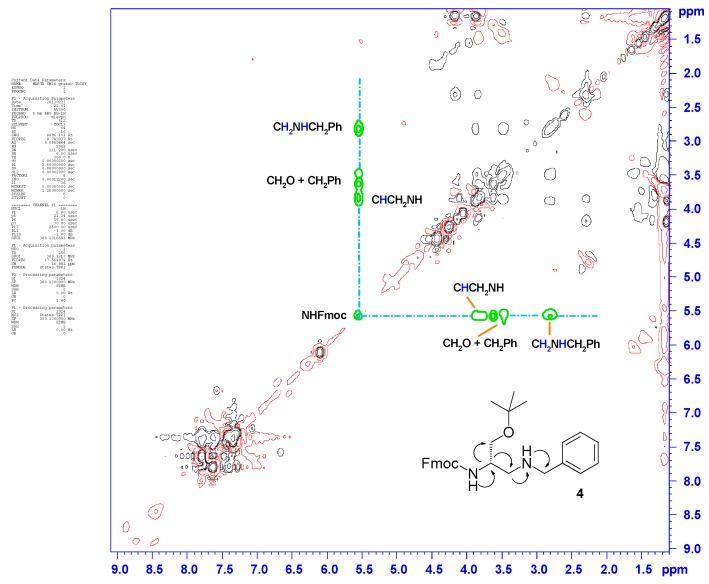
Contour plot of the two-dimensional (2D) homonuclear ^1^H,^1^H-TOCSY spectrum recorded for compound **4**.

The molecular structure of **4** was confirmed by one- and two-dimensional (1D and 2D) NMR spectroscopy. In particular, the 1D ^1^H- and ^13^C-NMR spectral plots displayed the correct number, multiplicity, and integral values of all resonances assignable to the expected structure of **4**. The contour plot of the 2D homonuclear proton TOCSY spectrum of the benzyl derivative **4** did not show signal overlap, facilitating the recognition of a set of cross-peaks at 5.52 ppm, in the regions between 3.90 and 3.40 ppm, and between 2.60 and 2.90 ppm, which were generated by the resonances of all correlated protons along the molecular backbone of **4** (Figure 1). Two-dimensional spectroscopy unequivocally proved the successful installation of the second amino function in substitution of the hydroxyl group of the starting d-serine backbone. These results highlighted that the reductive amination process can be a useful strategy for the realization of orthogonally protected 2,3-diaminopropanols.

The more sterically demanding diphenylmethylamine (benzhydrylamine) was a probe for observing the possible steric effects exerted by the nucleophile, as well as to prepare a 2,3-diaminopropanol scaffold containing the acid-labile benzhydryl masking group, which is orthogonal to the Fmoc protection [62]. Treatment of **3** with benzhydrylamine afforded the corresponding protected 2,3-diaminopropanol **5**, which was obtained in a 90% yield after chromatography. Results highlighted that the nucleophile size did not modify yields and reaction times. The proton NMR spectrum of **5** showed signal duplication, line broadening, and overlapping. Spectral data suggested the possible presence in solution of two main set of conformers in a 75:25 ratio, at the temperature fixed for NMR experiments. Coexistence of the two conformers is likely due to the slow rotation (on the NMR time-scale) around the amide bond of the carbamate residue. In carbamates, the two conformers *syn* and *anti* undergo dynamic exchange, with a general predominance of the *anti* isomer conformation with respect to its *syn* counterpart due to steric and electrostatic effects that can change the dipole moment and bond angles [63,64]. Solvent, temperature, and concentration can affect the free energy difference between the *syn* and *anti* conformers [65], and intra- and intermolecular hydrogen bonding may also perturb the *syn*/*anti* equilibrium [66]. In the case of compound **5**, the minor conformer was observable since it could be stabilized by the size and electronic effects of the benzhydryl group, as well as by the NMR solvent that could remarkably influence transition state energies and the rotational barrier in the syn/anti conformer exchange [67].

Alkylation of the amino functions is one of the most interesting modifications of l-Dap, and methylation of the 3-NH_2_ group is a structural feature displayed by many biologically active compounds [68,69]. Reductive amination of **3** with *n*-propylamine (Scheme 1, Table 1) gave **8** in an 89% yield after chromatography. The reaction represented a short route to the direct alkylation of the 3-NH_2_ function of the 2,3-diaminopropanol scaffold, and the *n*-propyl derivative **8** was considered to be a possible precursor in the synthesis of alkylated l-Dap structures.

### 3.3. Analysis of Racemization

**Figure 2:**
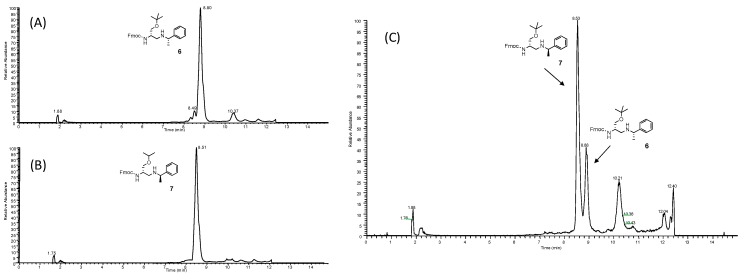
Reverse-phase (RP) U-HPLC/MS analysis of crude **6** (**A**), crude **7** (**B**), and a 2:1 mixture of diastereomers **6** and **7** (**C**).

Chiral α-amino aldehydes show the tendency to racemize under acidic or basic conditions, as well as during chromatographic purification over silica gel [70]. In order to check whether the chiral carbon atom configuration of the starting d-serine is preserved during the reductive amination process, the α-amino aldehyde **3** was reacted with the enantiomerically pure (*S*) and (*R*) isomers of 1-methylphenylamine. Reactions gave the corresponding diastereomeric 2,3-diaminopropanols **6** and **7** (Scheme 2, Table 1), which were obtained in a 90% and 91% yield, respectively, after chromatography. Both pure compounds **6** and **7** exhibited the expected molecular structure, as established by ^1^H- and ^13^C-NMR analysis and MS experiments. The NMR analysis was extended to the respective crude materials, as obtained from the reaction work-up, avoiding further purification steps. In this case, each NMR spectrum displayed signals which were diagnostically correlated to the proton and carbon resonances of a single diastereomer, with no evidence for signals attributable to other diastereomers, at least under the sensitivity limits of the spectrometric technique. Spectral data indicated that the Ti(IV)-assisted process of the imine intermediates was stereospecific [71]. Moreover, the absence of detectable resonance signals due to diastereomeric mixtures in each crude sample suggested that the reductive amination proceeded with no change of the configuration of the chiral carbon atom of **3**, consequently preserving the stereochemistry of the starting amino acid **1**. The absence of racemization was definitively confirmed by reverse-phase (RP) U-HPLC/MS analysis. Chromatograms recorded for separate injections of crude **6** and **7** showed a single elution peak for each compound (Figure 2).

### 3.4. Reductive Amination of the Aldehyde ***3*** with Sulfonamides

**Scheme 3:**
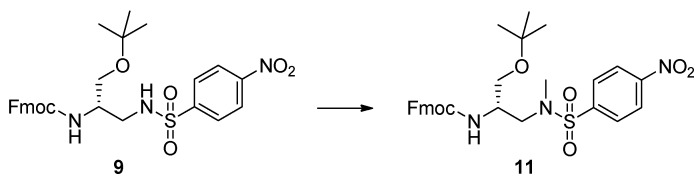
Methylation of **9**. Experimental conditions: (CH_3_O)_3_BF_4_/DIEA/DCM, 10 min, r.t. (quantitative yield).

We focused our attention on the reductive amination of **3** with *p*-toluenesulfonamide and 4-nitrobenzenesulfonamide (Scheme 2, Table 1). The required nucleophiles were prepared from the corresponding commercial sulfonyl chlorides, via reaction with a 20% ammonia solution. Reductive amination of **3** produced the arylsulfonyl-protected 2,3-diaminopropanols **9** and **10**, in very high yields (82% and 85%, respectively), after column chromatography. NMR spectroscopy and mass spectrometry experiments confirmed the structures of both compounds. The successive treatment of **9** with a slight excess of trimethyloxonium tetrafluoroborate [72,73] was site-specific, furnishing **11** as the unique product of methylation, in a quantitative yield, and without need for chromatography (Scheme 3).

Alternatively, methylation of **9** was realized using a threefold excess of diazomethane in dichloromethane, at room temperature. In this case, compound **11** was obtained, in short times, in quantitative yields, and avoiding chromatography. However, for a gram-scale preparation, diazomethane might be not the best option, because of its toxicity and dangerous preparation [74]. Compound **11** was reputed to be a possible intermediate for the synthesis of *N*^β^-methylated l-Dap scaffolds, after transformation of the OH function into a carboxyl group, followed by the final removal of the arylsulfonyl protection.

### 3.5. Synthesis of the l-Dap methyl Ester ***14***

The 2,3-aminopropanol **10** featured three orthogonal masking groups, a favorable characteristic for completing the l-Dap backbone realization. The presence of a tosyl group, which is largely employed in amino acid chemistry and peptide synthesis [75,76], convinced us to select **10** as the most likely candidate for the development of a short synthetic route to the desired l-Dap backbone. We designed an economical and mild method, which can limit chromatography and preserve sensitive masking groups.

**Scheme 4:**
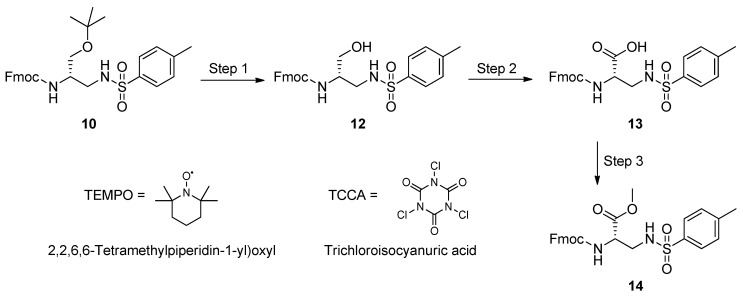
Oxidation of **10** with; 1,3,5-trichloro-2,4,6-triazinetrione (trichloroisocyanuric acid, TCCA)/ (2,2,6,6-tetramethylpiperidi-1-yl)oxyl (TEMPO): preparation of Fmoc-l-Dap(Ts)-OCH_3_ (**14**) Experimental conditions: Step 1 (acydolysis), Trifluoroacetic acid (TFA)/Trifluoroethanol (TFE)/DCM, 15 min, r.t. (quantitative yield); Step 2 (oxidation), TCCA/TEMPO/NaBr/aq. NaHCO_3_/Acetone, 20 min. at 0 °C, then overnight, r.t.; Step 3 (methylation), 0.66 N CH_2_N_2_ in DCM, 15 min, r.t. (**14**, yields: 92% over 2 steps, and 68% referred to the initial amount of **1**).

The synthetic procedure is depicted in Scheme 4. Initially, the 2,3-diaminopropanol **10** was reacted with trifluoroacetic acid (TFA) in the presence of trifluoroethanol (TFE). Acidolysis of the *tert*-butyl ether moiety generated the 2,3-diaminopropanol **12**, which was quantitatively obtained without need for chromatography. Compound **12** was then subjected to oxidation with 1,3,5-trichloro-2,4,6-triazinetrione; trichloroisocyanuric acid (TCCA) in the presence of catalytic amounts of (2,2,6,6-tetramethylpiperidi-1-yl)oxyl (TEMPO) and sodium bromide, to afford **13**. The crude carboxylic acid was finally treated with diazomethane in dichloromethane at room temperature, to give the l-Dap methyl ester **14**, which was obtained in an excellent overall yield, without chromatography.

### 3.6. Synthesis of the l-Dap Methyl Ester ***21***

**Scheme 5:**
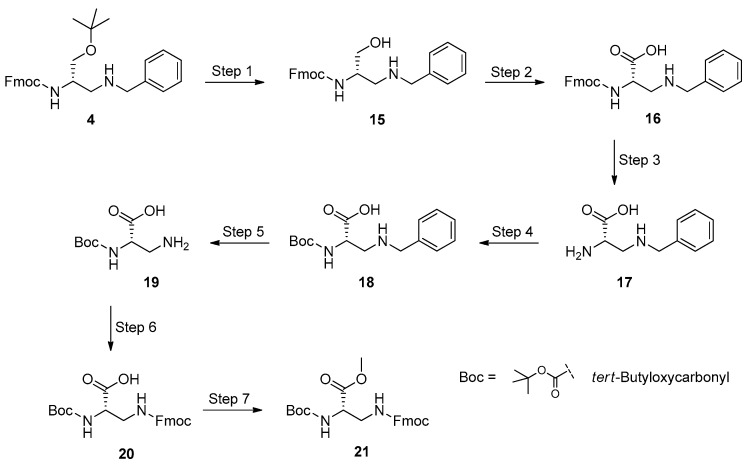
Multistep synthesis of Boc-l-Dap(Fmoc)-OH (**20**) and its derivative Boc-l-Dap(Fmoc)-OCH_3_ (**21**). Experimental conditions: Step 1 (acidolysis), TFA/TFE/DCM, 15 min, r.t.; Step 2 (oxidation), TCCA/TEMPO/NaBr/aq. NaHCO_3_/Acetone, 20 min at 0 °C, then overnight, r.t.; Step 3 (Fmoc removal), 20% DEA in DCM, 30 min, r.t.; Step 4 (protection with Boc), Boc_2_O/H_2_O/1,4-dioxane/NaHCO_3_, overnight, r.t.; Step 5 (hydrogenolysis), 1,4-cyclohexene/10% Pd-C/EtOH, overnight, 25 °C; Step 6 (protection with Fmoc), Fmoc-OSu/H_2_O/1,4-dioxane/NaHCO_3_, overnight, r.t.; Step 7 (methylation), 0.66 N CH_2_N_2_ in DCM, 15 min, r.t. (Yields: **20**, 93% over 6 steps, and 73% referred to the initial amount of **1**; **21**, quantitative from **20**).

Continually, we investigated the applicability of the TEMPO-mediated oxidation to the preparation of a l-Dap methyl ester, bearing the acid-sensitive Boc and the base-labile Fmoc group on the 2- and 3-NH_2_ functions, respectively. We were attracted by the idea of masking the side-chain amino function of l-Dap with the Fmoc group, because this protection can easily be removed under mild conditions during amino acid coupling sequences in solution phase and/or on solid supports, generally used in the total synthesis of oligopeptide strands and biologically active peptides. To fulfill our aim, we turned our attention on the benzyl-protected 2,3-diaminopropan-1-ol **4**. The presence of the benzyl group is beneficial, because it can effortlessly be removed by catalytic hydrogen transfer, preserving sensitive functional groups, base- and acid-labile masking moieties, and the chiral integrity of neighboring chiral carbon atoms. We planned a multi-step procedure, which could sequentially employ only crude products, as they are obtained via the simple work-up of the respective reactions, minimizing the number of time-expending and tedious chromatographic purifications. Scheme 5 displays the synthetic approach used to convert **4** into the targeted fully protected l-Dap methyl ester **21**.

The conversion of **4** into **20** began with the acidolysis of the *tert*-butyl ether linkage, to generate **15**. Unlike the synthesis of methyl ester **14**, we preferred to directly oxidize the crude intermediate 2,3-diaminopropanol **15**, without further purification. The reagent system TCCA/TEMPO/NaBr was used to obtain **16,** which was in turn treated with diethylamine (DEA) in dichloromethane, to remove the Fmoc protecting group. Unblocking of the 2-NH_2_ function afforded the carboxylic acid **17**, which was subsequently reacted with di-*tert*-butyl dicarbonate (Boc_2_O) to yield the Boc-derivative **18**. A catalytic transfer hydrogenation step [77] was applied to remove the benzyl residue from the 3-NH_2_ function of **18**, and the obtained carboxylic acid **19** was reacted with (*N*-(9-fluorenylmethoxycarbonyloxy)succinimide) (Fmoc-OSu), to afford the fully protected amino acid **20** in very high overall yields, after chromatography. ^1^H- and ^13^C-NMR spectroscopy showed all proton and carbon resonances as expected for the structure of **20**. At this point, we decided to check whether the chirality of the asymmetric carbon is preserved after the oxidation and unblocking/protection steps. Polarimetric measurements performed on a sample of pure **20** furnished an optical rotation similar to that obtained for a commercial specimen of the same compound, at the same concentration. The methyl ester **21** was quantitatively obtained by treating **20** with a slight excess of diazomethane in dichloromethane. NMR spectroscopy confirmed the molecular structure of **21**, and its optical rotation was almost equal to that reported in the literature for the same optically pure compound prepared by a different procedure [51], within experimental error. This finding unambiguously proved that racemization does not occur in our synthesis of l-Dap, preserving the chirality of **1** throughout the applied multistep procedure.

### 3.7. Couplings of l-Dap Methyl Ester ***21***

**Scheme 6:**
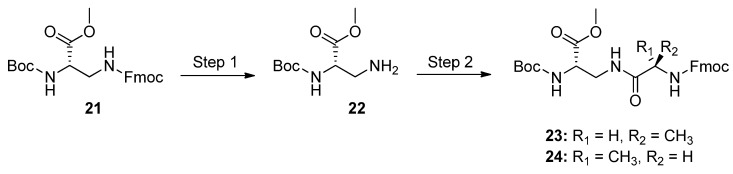
Side-chain elongation of **21**: preparation of dipeptides **23** and **24**. Experimental conditions: Step 1 (Fmoc removal), 20% DEA in DCM, 30 min, r.t.; Step 2 (coupling): Fmoc-l-Ala-OH or Fmoc-d-Ala-OH, HOBt monohydrate/EDC/DIEA/DCM, overnight, r.t.

As the conclusive point of our study, the l-Dap methyl ester **21** was subjected to couplings with both enantiomerically pure l and d optical isomers of Fmoc-Ala-OH, to obtain dipeptides **23** and **24** (Scheme 6).

This experiment was performed in order to verify the possible use of **21** in solution-phase Fmoc-chemistry-based peptide synthesis, and to check whether racemization of **21** occurs during the coupling process. Diastereomers **23** and **24** were prepared via a classical solution-phase strategy [78]. Accordingly, **21** was treated with DEA to unblock the 3-NH_2_ group, and then separately coupled to both *N*-Fmoc-alanine enantiomers. The system 1-(3-dimethylaminopropyl)-3-ethylcarbodiimide hydrochloride/1-hydroxybenzotriazole (EDC/HOBt) was adopted to activate the carboxyl function of both protected alanine enantiomers, in the presence of the Hunig’s base (diisopropylethylamine, DIEA). Reactions gave dipeptides **23** and **24**, which were directly analyzed by RP U-HPLC/MS (see Supplementary Materials), without pre-injection purification. Instrumental analysis highlighted that only one product was obtained from each coupling, confirming that the experimental conditions adopted for the side-chain elongation of **21** proceeded without detectable changes of its chirality.

## 4. Materials and Methods

Chemicals and solvents were reagent and HPLC grade (Sigma-Aldrich, Milano, Italy); amino acids were purchased from Senn Chemicals (Bülack, Switzerland). All reagents were used without further purification. Solvents (Sigma-Aldrich, Milano, Italy) were purified according to standard procedures, and freshly distilled under a dry nitrogen current, prior to use. Petroleum ether 60–80 °C was used for TLC and column chromatography. Melting points were determined by a Kofler hot-stage apparatus (Reichert-Thermovar, Germany), at atmospheric pressure, and they are uncorrected. Optical rotations were measured in a 1-dm^3^ (2-mL volume) tube, in methanol (c 1.0), and [α]_D_ values were determined at 20 °C, using a digital polarimeter. All NMR spectra were recorded at 25 ± 0.1 °C, using 5-mm tubes (seven inches in length), on an Avance 300 Ultrashielded Spectrometer (Bruker Biospin, Milano, Italy), equipped with a BB0 probe and a temperature control unit. One-dimensional ^1^H- and ^13^C-NMR spectra were recorded at 300.132 MHz and 75.03 MHz, respectively. Deuterated chloroform for spectroscopy (CDCl_3_, 99.5% isotopically pure, containing 0.1% tetramethylsilane (TMS)) was used as the solvent for all spectral analysis. Chemical shifts (δ) are reported in parts per million (ppm), and they are referred to the resonance of the residual proton of the solvent (singlet at 7.25 ppm) and to the central line (77.0 ppm) of the solvent triplet, in proton and carbon spectra, respectively. Proton coupling constants (*J*) are reported in Hz. Relaxation times (T_1_) for all ^1^H and ^13^C carbon nuclei were calculated experimentally for all synthesized compounds, using a pulse sequence and method as published in the literature [79] and fixed to the superior limits of 3 s (proton) and 10 s (carbon). Extensive proton decoupling experiments were applied to unequivocally assign all resonances to the respective protons. Two-dimensional homonuclear correlation spectra (^1^H,^1^H-TOCSY) were obtained by the M.Levitt’s composed pulse decoupling sequence (MLEV) for isotropic conditions, with an 80 ms spin-lock period, elaborating spectral data as previously reported [80]. Acquisition and elaboration parameters for one-dimensional ^1^H and ^13^C spectra were as already published [81]. Reverse-phase ultrafast HPLC/mass spectrometry (RP U-HPLC/MS) was carried out using a Vantage RP U-HPLC instrument (Thermo), coupled to a triple quadrupole mass spectrometer and equipped with a heated electrospray ion source (HESI II), which operated in positive mode [82]. Chromatographic runs were obtained on a C-18 RP analytical column (Hypersyl Gold, 2.1 × 50 mm, particle size 1.9 μm), under isocratic conditions and with a 0.4 mL/min flux, applying a 55:45 water (containing 0.1% TFA)/methanol elution gradient. ESI(+) MS spectra were obtained by a Q-star *i*-pulsar instrument (AB Sciex), operated in positive mode at 20 keV, and equipped with a TOF ion analyzer [82]. HRMS analyses were performed using a MALDI-TOF/TOF 5800 spectrometer (AB Sciex), in reflectron positive mode [83,84]. Reactions were monitored by thin-layer chromatography (TLC), using pre-coated silica gel glass plates (Merck, 60-F254, 0.25 mm). The best elution of carboxylic acids was obtained by adding glacial acetic acid (three drops) to the eluent mixture (5 mL). Chromatographic spots were visualized with an ultraviolet (UV) lamp (254 nm), ninhydrine (0.2% in *n*-butanol), vanilline solution (5% in ethanol/H_2_SO_4_), and H_2_SO_4_ (20% in absolute ethanol). After elution, charring at 200 °C was applied to develop spot colorations. Kieselgel 60H without gypsum (Merck) was used as the stationary phase for short-column flash chromatography, adopting the same gradients used for the respective TLC analyses, with the polar component being reduced four-fold. All reactions were carried out with pre-flamed glassware and under an inert atmosphere (dry N_2_). Diazomethane in DCM (0.66 M solution) was freshly prepared starting from *N*-methyl-*N*-nitrosourea, accordingly to the published procedure [74], and then stored on KOH pellets at −20 °C. Diazomethane concentration was obtained by back-titration with a standard solution of benzoic acid.

### 4.1. Synthesis of the Weinreb–Nahm Amide ***2***

HOBt monohydrate (239.6 mg, 1.57 mmol), DIC (0.24 mL, 1.57 mmol), and DIEA (0.27 mL, 1.57 mmol) were added to a solution of *N*^α^-Fmoc-*O*-*tert*-butyl-d-serine (**1**; 500 mg, 1.30 mmol) in dry DCM (5 mL), under magnetic stirring. After 2 h at room temperature, a solution of *N,O*-dimethylhydroxylamine hydrochloride (127.2 mg, 1.304 mmol) and DIEA (1.2 equivalents, 0.27 mL, 1.57 mmol) in dry DCM (5 mL) was added dropwise, and the resulting mixture was maintained under magnetic stirring overnight at room temperature (TLC: diethyl ether/petroleum ether, 80:20 *v*/*v*). The solvent was removed under reduced pressure condition, and the recovered solid residue was re-dissolved in 5% aqueous NaHSO_4_ (10 mL), then extracted with diethyl ether (3 × 10 mL). The organic layers were washed with 5% aqueous NaHCO_3_ (3 × 10 mL) and brine (3 × 10 mL), dried over Na_2_SO_4_, paper filtered, and evaporated to dryness under reduced pressure conditions. The crude product was purified by flash column chromatography (FCC) eluent: diethyl ether/petroleum ether, 95:5 *v*/*v*), to obtain the title product. White solid (522.7 mg, 94%), mp 136–138 °C; ^1^H-NMR (300 MHz, CDCl_3_) δ 7.75 (d, 2H, *J* = 7.0, ArH Fmoc), 7.61 (d, 2H, *J* = 7.0, ArH Fmoc), 7.38 (t, 2H, *J* = 7.0, ArH Fmoc), 7.30 (t, 2H, *J* = 7.0, ArH Fmoc), 5.86 (d, 1H, *J* = 7.2, NH), 4.88 (bs, 1H, C*H*NH), 4.36 (d, 2H, *J* = 6.9, CH_2_ Fmoc), 4.23 (t, 1H, *J* = 6.9, CH Fmoc), 1.16 (s, 9H, CH_3_ *t*Bu); ^13^C-NMR (75 MHz, CDCl_3_) δ 170.6, 155.9, 143.7, 141.1, 127.5, 126.9, 115.0, 119.7, 73.4, 66.9, 61.8, 61.3, 51.6, 46.9, 32.0, 27.1; MALDI HRMS: calculated 427.2227 for C_24_H_31_N_2_O_5_^+^, found 427.2244.

### 4.2. Synthesis of the Aldehyde ***3***

A solution of **2** (522,7 mg, 1.23 mmol) in dry THF was treated with LiAlH_4_ (93.1 mg, 2.45 mmol), under magnetic stirring at room temperature. Reaction was complete in 12 min, as checked by TLC (diethyl ether/petroleum ether 70:30, *v*/*v*). A 5% aqueous solution of NaHCO_3_ was added (CAUTION), and the mixture was stirred for 10 min. The ethereal phase was separated, and the aqueous residue was washed with fresh diethyl ether (3 × 10 mL). The organic extracts were collected, washed with a 5% aqueous solution of NaHSO_4_ (3 × 10 mL) and once with brine (10 mL), dried over Na_2_SO_4_, paper-filtered, and evaporated to dryness under reduced pressure conditions, to give **3**. The aldehyde was pure enough and was used in the next step without further purification. Colorless oil (415.3 mg, 92%); TLC (diethyl ether/petroleum ether 70:30, *v*/*v*): R*_f_* = 0.65; ^1^H-NMR (300 MHz, CDCl_3_) δ 9.63 (d, 1H, *J* = 2.8, CHO), 7.75 (d, 2H, *J* = 7.0, ArH), 7.62 (d, 2H, *J* = 7.0, ArH), 7.40 (t, 2H, *J* = 7.0, ArH), 7.32 (t, 2H, *J* = 7.0, ArH), 5.71 (d, 1H, *J* = 6.0, NH), 4.42 (d, 2H, *J* = 7.1, CH_2_ Fmoc) 4.32-4.38 (m, 1H, C*H*NH), 4.25 (t, 1H, *J* = 7.1, CH Fmoc), 3.96 (dd, 1H, *J* = 11.2 and 7.6, CH_2_O), 3.63 (dd, 1H, *J* = 11.2 and 5.8, CH_2_O), 1.16 (s, 9H, CH_3_); ^13^C-NMR (75 MHz, CDCl_3_) δ 199.1, 156.1, 143.7, 141.2, 127.7, 127.0, 125.0, 119.9, 73.7, 67.1, 60.4, 59.9, 47.0, 27.1; MALDI HRMS: calculated for C_22_H_26_NO_4_^+^ 368.1858, found 368.1869.

### 4.3. Synthesis of ***4***–***10***. General Procedure for the Reductive Amination

To a solution of **3** (206.6 mg, 0.56 mmol) in dry ethanol (4 mL), Ti(OiPr)_4_ (0.34 mL, 1.12 mmol) was added under magnetic stirring at room temperature. After 10 min, the appropriate primary amine or arylsulfonylamide (0.67 mmol) was added. The resulting mixture was maintained under stirring at room temperature until complete conversion of **3**, monitoring the imine formation by TLC (diethyl ether/petroleum ether, 80:20 *v*/*v*), and NaBH_3_CN (52.8 mg, 0.84 mmol) was added. The mixture was stirred overnight at room temperature, and brine (5 mL) was then added. The white precipitate was filtered off by paper filtration and washed with a minimum amount of absolute ethanol. The mother liquor was concentrated under reduced pressure conditions, and the residue was eluted with ethyl acetate through a short pad of Celite 545^®^. The organic solution was dried over MgSO_4_, paper-filtered, and evaporated to dryness under vacuum, to give a crude material that was fractioned by column chromatography (eluent: AcOEt/*n*-hexane, 50:50 *v*/*v*, reducing the polar component four-fold), to deliver the desired 2,3-diaminopropanols **4**–**10** in an 82%–92% overall yield.

*2,3-Diaminopropanol***4**. Colorless oil (236.9 mg, 92%); TLC (diethyl ether/*n*-hexane 80:20, *v*/*v*): R*_f_* = 0.17; ^1^H-NMR (300 MHz, CDCl_3_) δ 7.76 (d, 2H, *J* = 7.0, ArH Fmoc), 7.60 (d, 2H, *J* = 7.0, ArH Fmoc), 7.28–7.43 (m, 9H, ArH Fmoc and ArH phenyl), 5.52 (d, 1H, *J* = 6.8, NHCO), 4.37 (d, 2H, *J* = 6.9, CH_2_ Fmoc), 4.22 (1H, *J* = 6.9, CH Fmoc), 3.77–3.92 (m, 4H, CH, CH_2_O and PhCH_2_), 3.41–3.49 (m, 1H, CH_2_O), 2.73–2.81 (m, 2H, CH_2_N), 2.25–2.40 (m, 1H, NH), 1.19 (s, 9H, CH_3_ *t*Bu); ^13^C-NMR (75 MHz, CDCl_3_) δ 156.6, 144.1, 141.2, 136.0, 128.7, 127.93, 127.91, 126.6, 124.7, 124.2, 119.8, 73.7, 68.5, 66.8, 55.4, 52.8, 48.0, 47.1, 27.1; ESI(+) MS: *m/z* 459.38 [M + H]^+^; MALDI HRMS: calculated for C_29_H_35_N_2_O_3_^+^ 459.2642, found 459.2658.

*2,3-Diaminopropanol***5.** Pale yellow oil (280.2 mg, 90%); TLC (diethyl ether/*n*-hexane 80:20, *v*/*v*): R*_f_* = 0.25; ^1^H-NMR (300 MHz, CDCl_3_) severe overlap, 75:25 mixture of two conformers, δ 7.79 (d, 4H, *J* = 6.9, ArH Fmoc), 7.65 (d, 4H, *J* = 6.9, ArH Fmoc), 7.19–7.49 (m, 28H, ArH Fmoc and ArH phenyls), 5.76 (d, 0.25H, *J* = 8.9, NHCO minor conformer), 5.55 (d, 0.75H, *J* = 7.2, NHCO major conformer), 4.86 (sb, 2H, CHPh_2_), 4.44–4.48 (m, 0.25H, C*H*NH minor conformer), 4.42 (d, 4H, *J* = 8.0, CH_2_ Fmoc), 4.22 (t, 2H, *J* = 8.0, CH Fmoc), 4.09–4.16 (m, 0.75H, C*H*NH major conformer), 3.81–3.86 (m, 0.50H, CH_2_O minor conformer), 3.45–3.69 (m, 1.5H, CH_2_O major conformer), 2.70–2.80 (m, 2H, CH_2_N major conformer), 2.12–2.39 (m, 4H, CH_2_N minor conformer, and NH both conformers), 1.17 (s, 18H, CH_3_ *t*Bu); ^13^C-NMR (75 MHz, CDCl_3_) δ 156.1, 155.8, 145.3, 144.1, 143.9, 143.7, 143.6, 143.0, 141.2, 128.7, 128.6, 128.4, 127.7, 127.6, 127.5, 127.2, 127.1, 126.90, 126.88, 126.81, 125.0, 124.7, 120.0, 119.9, 73.8, 72.9, 67.2, 67.0, 66.6, 50.8, 50.5, 49.1, 47.2, 47.0, 27.3, 27.2; ESI(+) MS: *m/z* 557.72 [M + H]^+^; MALDI HRMS: calculated for C_39_H_38_N_2_NaO_3_^+^ 557.2775, found 557.2797.

*2,3-Diaminopropanol***6**. Colorless oil (237.9 mg, 90%); TLC (diethyl ether/*n*-hexane 80:20, *v*/*v*): R*_f_* = 0.21; ^1^H-NMR (300 MHz, CDCl_3_) δ 7.76 (d, 2H, *J* = 7.2 Hz, ArH Fmoc), 7.61 (d, 2H, *J* = 7.2 Hz, ArH Fmoc), 7.40 (t, 2H, *J* = 7.2 Hz, ArH Fmoc), 7.20–7.40 (m, 7H, ArH Fmoc and ArH phenyl), 5.43 (d, 1H, *J* = 7.9, NHCO), 4.38-4.48 (m, 1H, CH Fmoc), 4.21–4.36 (m, 2H, CH_2_ Fmoc), 3.68–3.82 (m, 2H, CH_2_O and CH_2_C*H*NH), 3.54–3.97 (m, 1H, C*H*NH), 2.60–2.76 (m, 1H, C*H*_2_NH), 2.23–2.46 (m, 2H, C*H*_2_NH and CHN*H*), 1.34 (d, 3H, *J* = 6.5, CH_3_), 1.14 (s, 9H, CH_3_ *t*Bu); ^13^C-NMR (75 MHz, CDCl_3_) δ 156.3, 145.8, 143.4, 141.3, 129.4, 128.4, 127.6, 127.5, 127.0, 126.5, 125.0, 119.9, 73.2, 66.9, 62.2, 55.2, 47.4, 42.1, 27.5, 23.4; ESI(+) MS: *m/z* 473.42 [M + H]^+^; MALDI HRMS: calculated for C_30_H_37_N_2_O_3_^+^ 473.2799, found 473.2817.

*2,3-Diaminopropanol***7**. Colorless oil (240.5 mg, 91%); TLC (diethyl ether/*n*-hexane 80:20, *v*/*v*): R*_f_* = 0.21; ^1^H-NMR (300 MHz, CDCl_3_) δ 7.77 (d, 2H, *J* = 7.3, ArH Fmoc), 7.60 (d, 2H, *J* = 7.3, ArH Fmoc), 7.40 (t, 2H, *J* = 7.3, Ar Fmoc), 7.19–7.39 (m, 7H, ArH Fmoc and ArH phenyl), 5.52 (d, 1H, *J* = 7.8, NHCO), 4.46–4.21 (m, 3H, CH_2_ and CH Fmoc), 3.63–3.92 (m, 2H, CH_2_O), 3.55–3.62 (m, 1H, C*H*CH_2_N and C*H*NH), 2.50–2.72 (m, 3H, CH_2_N and CHN*H*), 1.16 (d, 3H, *J* = 6.8, CH_3_), 1,13 (s, 9H, CH_3_ *t*Bu); ^13^C-NMR (75 MHz, CDCl_3_) δ 157.1, 145.8, 144.0, 141.3, 129.4, 128.7, 127.6, 127.5, 126.4, 126.4, 125.3, 115.0, 73.2, 63.0, 53.5, 50.5, 48.8, 47.3, 27.3, 24.2; ESI(+) MS: *m/z* 473.35 [M + H]^+^; MALDI HRMS: calculated for C_30_H_37_N_2_O_3_^+^ 473.2799, found 473.2780.

*2,3-Diaminopropanol***8**. Colorless oil (204.3 mg, 89%); TLC (diethyl ether/*n*-hexane 80:20, *v*/*v*): R*_f_* = 0.14; ^1^H-NMR (300 MHz, CDCl_3_) δ 7.65–7.80 (m, 2H, ArH), 7.61 (d, 2H, *J* = 7.0, ArH), 7.26-7.45 (m, 4H, ArH), 5.63 (bs, 1H, NHCO), 4.38 (d, 2H, *J* = 6.9, CH_2_ Fmoc), 4.23 (1H, *J* = 6.9, CH Fmoc), 3.31–3.45 (m, 3H, CH and CH_2_O), 2.51–2.90 (m, 4H, CH_2_N), 1.30-1.58 (m, 2H, C*H*_2_CH_3_), 1.16 (s, 9H, CH_3_ *t*Bu), 0.75–0.90 (m, 3H, C*H*_3_CH_2_); ^13^C-NMR (75 MHz, CDCl_3_) δ 156.2, 143.7, 141.3, 127.8, 127.2, 125.0, 124.8, 119.9, 75.6, 67.5, 52.0, 48.3, 27.2, 21.6, 11.9; ESI(+) MS: *m/z* 411.29 [M + H]^+^; MALDI HRMS: calculated for C_25_H_35_N_2_O_3_^+^ 411.2642, found 411.2660.

*2,3-Diaminopropanol***9**. Pale yellow solid (253.9 mg, 82%); TLC (diethyl ether/*n*-hexane 80:20, *v*/*v*): R*_f_* = 0.24; mp 127–129 °C; ^1^H-NMR (300 MHz, CDCl_3_) δ 8.20–8.34 (m, 2H, SO_2_ArH), 7.90–8.10 (m, 2H, SO_2_ArH), 7.76 (d, 2H, *J* = 7.2, ArH Fmoc), 7.55 (d, 2H, *J* = 7.2, ArH Fmoc), 7.33–7.44 (m, 2H, ArH Fmoc), 7.20–7.32 (m, 2H, ArH Fmoc), 5.88 (bs, 1H, NHSO_2_), 5.32 (d, 1H, *J* = 7.6, NHCO), 4.35–4.49 (m, 1H, C*H*NH), 4.29–4.35 (m, 2H, CH_2_ Fmoc), 4.10–4.19 (m, 1H, CH Fmoc), 4.00–4.09 (m, 1H, CH_2_O), 3.73–3.87 (m, 1H, CH_2_O), 3.15–3.31 (m, 1H, CH_2_N), 3.00–3.15 (m, 1H, CH_2_N), 1.16 (s, 9H, CH_3_ *t*Bu); ^13^C-NMR (75 MHz, CDCl_3_) δ 156.4, 150.0, 145.9, 143.7, 141.3, 128.2, 127.8, 127.0, 124.9, 124.3, 120.0, 73.9, 67.1, 62.3, 50.1, 47.1, 42.1, 27.3; ESI(+) MS: *m/z* 554.38 [M + H]^+^; MALDI HRMS: calculated for C_28_H_32_N_3_O_7_S^+^ 554.1955, found 554.1938.

*2,3-Diaminopropanol***10**. White solid (239.7 mg, 85%); TLC (diethyl ether/*n*-hexane 80:20, *v*/*v*): R*_f_* = 0.22; mp 119–121 °C; ^1^H-NMR (300 MHz, CDCl_3_) δ 7.68-7.80 (m, 4H, SO_2_ArH), 7.58 (d, 2H, *J* = 7.4, ArH Fmoc), 7.40 (t, 2H, *J* = 7.4, ArH Fmoc), 7.23–7.35 (m, 4H, SO_2_ArH and ArH Fmoc), 5.32–5.48 (m, 2H, NHCO and NHSO_2_), 4.33 (d, 2H, *J* = 7.2, CH_2_ Fmoc), 4.19 (t, 1H, *J* = 7.2, CH Fmoc), 3.75–3.85 (m, 1H, C*H*NH), 3.40–3.50 (m, 2H, CH_2_O), 3.16-3.28 (m, 1H, CH_2_N), 3.00–3.11 (m, 1H, CH_2_N), 2.37 (s, 3H, CH_3_ tosyl), 1.15 (s, 9H, CH_3_ *t*Bu); ^13^C-NMR (75 MHz, CDCl_3_) δ 156.2, 141.2, 136.7, 129.7, 127.8, 127.5, 127.0, 125.1, 124.7, 120.0, 73.7, 66.9, 65.0, 50.3, 49.7, 47.1, 45.6, 27.3, 21.4; ESI(+) MS: *m/z* 523.65 [M + H]^+^; MALDI HRMS: calculated for C_29_H_35_N_2_O_5_S^+^ 523.2261, found 523.2287.

### 4.4. Methylation of ***9***. Synthesis of 2,3-diaminopropanol ***11***

Compound **9** (100.0 mg, 0.18 mmol) was methylated with trimethyloxonium tetrafluoroborate (66.4 mg, 0.45 mmol), in the presence of DIEA (0.11 mL, 0.63 mmol), in DCM (20 mL), accordingly to the previously published procedure [76]. Pale yellow oil (102.1 mg, quantitative); TLC (diethyl ether/*n*-hexane 80:20 *v*/*v*) R*_f_* = 0.48; ^1^H-NMR (300 MHz, CDCl_3_) δ 8.29–8.44 (m, 2H, SO_2_ArH), 7.91–8.09 (m, 2H, SO_2_ArH), 7.70–7.80 (m, 2H, ArH Fmoc), 7.50-7.65 (m, 2H, ArH Fmoc), 7.31–7.42 (m, 2H, ArH Fmoc), 7.23–7.31 (m, 2H, ArH Fmoc), 5.34 (d, 1H, *J* = 7.1, C*H*NH), 4.30–4.41 (m, 2H, CH_2_ Fmoc), 4.12-4.28 (m, 1H, CH Fmoc), 3.75–3.97 (m, 1H, C*H*NH), 3.60–3.73 (m, 1H, CH_2_O), 3.32–3.48 (m, 2H, CH_2_O and CH_2_N), 3.10-3.21 (m, 1H, CH_2_N), 2.83 (s, 3H, CH_3_), 1.16 (s, 9H, CH_3_ *t*Bu); ^13^C-NMR (75 MHz, CDCl_3_) δ 156.1, 150.0, 143.6, 141.2, 127.7, 127.0, 125.0, 124.7, 124.4, 120.0, 73.5, 66.9, 60.5, 54.7, 51.0, 49.1, 47.1, 35.7, 27.2; ESI(+) MS: *m/z* 568.19 [M + H]^+^; MALDI HRMS: calculated for C_29_H_34_N_3_O_7_S^+^ 568.2112, found 568.2133.

### 4.5. Synthesis of 2,3-diaminopropanol ***12***

To a solution of **10** (100.0 mg, 0.19 mmol), in dry DCM (3 mL), TFA (1 mL) and TFE (0.3 mL) were added. The reaction mixture was magnetically stirred at room temperature for 15 min (TLC: diethyl ether/petroleum ether 80:20, *v*/*v*). After complete conversion of **10**, the mixture was evaporated to dryness under reduced pressure conditions. The residue was dissolved in DCM (5 mL), treated with solid Na_2_CO_3_ (CAUTION) until complete gas evolution, and then desalted by elution with small volumes of DCM on a short silica gel pad. The organic solution was dried over Na_2_SO_4_, and evaporated to dryness under reduced pressure conditions, to afford **12** (88.5 mg, quantitative). White solid, mp 142–145 °C; TLC (diethyl ether/*n*-hexane 80:20, *v*/*v*): R*_f_* = 0.16; ^1^H-NMR (300 MHz, CDCl_3_) δ 7.62–7.80 (m, 4H, ArH Fmoc and ArH Ts), 7.45–7.60 (m, 2H, ArH Fmoc), 7.30–7.40 (m, 2H, ArH Fmoc), 7.19–7.30 (m, 4H, ArH Fmoc and ArH Ts), 5.52–5.73 (m, 1H, NHCO), 5.18 (bs, 1H, NHSO_2_), 4.00–4.44 (m, 4H, CH Fmoc, CH_2_ Fmoc and C*H*NH), 3.52–3.78 (m, 2H, CH_2_O), 3.01 (bs, 1H, OH), 2.50–2.71 (m, 2H, C*H*_2_NH), 2.32 (s, 3H, CH_3_); ^13^C-NMR (75 MHz, CDCl_3_) δ 156.6, 143.5, 141.2, 139.0, 136.4, 129.8, 127.7, 123.1, 126.3, 125.1, 119.9, 67.0, 62.9, 53.4, 47.0, 43.2, 21.4; MALDI HRMS: calculated for C_25_H_27_N_2_O_5_S^+^ 467.1635, found 467.1659.

### 4.6. Preparation of Fmoc-l-Dap(Ts)-OCH_3_ *(****14****)*

Compound **12** (232.8 mg, 0.5 mmol) was dissolved in acetone (5 mL), and aqueous 15% solution of NaHCO_3_ (3 mL) was added, followed by solid NaBr (10.2 mg, 0.1 mmol). TEMPO (7.5 mg, 0.05 mmol) was added to the mixture, under magnetic stirring at 0 °C. After slow addition of TCCA (220.5 mg, 5.0 mmol) within 20 min at 0 °C, the mixture was maintained under magnetic stirring overnight at room temperature. Then, 2-propanol (3 mL) was added, and the mixture was filtered on a short pad of Celite 545^®^, concentrated under vacuum, and treated with 5 mL of a saturated aqueous solution of Na_2_CO_3_. The aqueous phase was washed with small portions of AcOEt (3 × 10 mL), treated with 5% aqueous KHSO_4_ (10 mL), and re-extracted with AcOEt (3 × 10 mL). The organic layers were dried over MgSO_4_ and paper-filtered, and the solvent was evaporated to dryness, yielding a raw material which was directly treated with an excess of diazomethane (0.66 N solution in dry DCM, 4 mL), under magnetic stirring for 15 min at room temperature [85]. The mixture was evaporated to dryness under reduced pressure conditions (CAUTION: use a fume hood), to give **14**. White solid (227.2 mg; 92%, over 2 steps; 68% total yield, based on the initial amount of the amino acid **1**, 1.30 mmol); mp >90 °C, dec.; TLC (diethyl ether/*n*-hexane 80:20, *v*/*v*) R*_f_* = 0.36; ^1^H-NMR (300 MHz, CDCl_3_) δ 7.65–7.79 (m, 4H, Ar Fmoc and ArH Ts), 7.52–7.61 (m, 2H, ArH Fmoc), 7.35–7.43 (m, 2H, ArH Fmoc), 7.22–7.34 (m, 5H, Ar Fmoc, ArH Ts and NHSO_2_), 5.89 (d, 1H, *J* = 6.5 Hz, NHCO), 4.48-4.62 (m, 4H, CH Fmoc, CH_2_ Fmoc and C*H*NH), 3.95 (dt, 1H, *J* = 21 and 11 Hz, CH_2_N), 3.94 (dt, 1H, *J* = 21 and 11 Hz, CH_2_N), 3.77 (s, 3H, OCH_3_), 2.38 (s, 3H, CH_3_); ^13^C-NMR (75 MHz, CDCl_3_) δ 171.1, 156.8, 143.6, 141.3, 123.7, 129.6, 128.6, 127.7, 127.0, 126.4, 125.0, 119.9, 67.1, 56.0, 52.7, 47.1, 41.8, 22.1; MALDI HRMS: calculated for C_26_H_27_N_2_O_6_S^+^ 495.1584, found 495.1606.

### 4.7. Synthesis of Boc-l-Dap(Fmoc)-OH *(****20****)*: Multistep Procedure

Step 1: Acidolysis. To a solution of **4** (229 mg, 0.5 mmol), in dry DCM (3 mL), TFA (2 mL) and TFE (0.3 mL) were added. The reaction mixture was magnetically stirred at room temperature for 15 min (TLC: diethyl ether/petroleum ether 80:20, *v*/*v*). After complete conversion of **4**, the mixture was evaporated to dryness under reduced pressure conditions. The residue was dissolved in DCM (5 mL), treated with solid Na_2_CO_3_ until complete gas evolution, and desalted on a short pad of Celite 545^®^ by elution with small volumes of DCM. The organic phase was dried over Na_2_SO_4_ and evaporated to dryness under reduced pressure conditions, to afford a crude solid which was directly used in the next step, without purification. Step 2: Oxidation. The crude material obtained from the previous step was subjected to oxidation, applying the procedure above described for the preparation of the methyl ester **14**. The final crude product was dried under vacuum, and then subjected to the next step without further purification. Step 3: Removal of Fmoc group. The crude product recovered from step 2, was reacted with 5 mL of a 20% DEA solution in DCM, under magnetic stirring at room temperature for 30 min. Evaporation to dryness of volatile gave a solid residue that was dissolved in AcOEt and precipitated with cold n-pentane. The solid was filtered, washed three times with small portions of n-pentane, and dried under vacuum. The crude material, as recovered, was immediately subjected to the next step of the procedure. Step 4: Protection with Boc group. The crude solid recovered from step 3 was suspended in H_2_O (5 mL), containing NaHCO_3_ (two equivalents with respect to **4**; 88 mg, 1 mmol), and a solution of di-*tert*-butyl-dicarbonate (1.1 equivalents with respect to **4**; 120 mg, 0.55 mmol) in 1,4-dioxane (5 mL) was added dropwise to the reaction system. The resulting clear mixture was maintained under stirring overnight at room temperature, and then concentrated under reduced pressure conditions to give an aqueous residue which was made acidic by adding 5% aqueous KHSO_4_, before being extracted with ethyl acetate (3 × 10 mL). The organic layers were collected, washed once with brine (10 mL), dried over Na_2_SO_4_, paper-filtered, and evaporated to dryness to give a white solid which was transformed in a white powder by co-evaporation from a 2:1 (*v*/*v*) methyl-*tert*-butyl ether (MTB)/*n*-hexane mixture. TLC (CHCl_3_/MeOH 90:10, *v*/*v*): R*_f_* = 0.34. Step 5: Hydrogenolysis of benzyl group. The crude product recovered from step 4 was dissolved in absolute ethanol (5 mL), and the reaction flask was immersed in a water bath at 25 °C. To the alcoholic solution, 10% palladium-carbon (22.9 mg) and 1,4-cyclohexadiene (10 equivalents with respect to **4**; 0.47 mL, 5.0 mmol) were added, and the mixture was allowed to react under magnetic stirring overnight. After filtration over a short pad of Celite 545^®^, the solvent was evaporated under reduced pressure conditions, and the solid residue was partitioned in a 1:1 (*v*/*v*) H_2_O/EtOAc mixture (10 mL). The aqueous phase was separated, back extracted with EtOAc (3 × 10 mL), concentrated under reduced pressure conditions, and lyophilized. Step 6: Protection with Fmoc group. The crude product obtained from step 5 was dissolved in a 1:1 (*v*/*v*) 1,4-dioxane/H_2_O mixture (15 mL) containing NaHCO_3_ (two equivalents with respect to **4**; 88 mg, 1 mmol). A solution of Fmoc-OSu (1.1 equivalents with respect to **4**; 185.5 mg, 25 mmol) in 1,4-dioxane (5 mL) was added dropwise in 35 min, and the resulting mixture was magnetically stirred overnight at room temperature. The reaction was checked by TLC (CHCl_3_/MeOH 95:5, *v*/*v*; ninhydrin as the visualizing agent), and, after complete disappearing of the free amino compound, the solution was concentrated under vacuum, and the aqueous residue was made acidic (pH = 2.5) by adding 5% aqueous KHSO_4_ and extracted with ethyl acetate (3 × 10 mL). The collected organic layers were washed once with brine (10 mL), dried over Na_2_SO_4_, paper=filtered, and evaporated to dryness to give a viscous residue which was rapidly dissolved in the smallest volume of DCM and precipitated from cold *n*-pentane. The precipitate was filtered under vacuum and dried by using an oil pump. The final product was purified by column chromatography. White powder (404.7 mg; 93% over six steps; 73% based on the initial amount of **1**, 1.30 mmol); mp >78 °C (dec.); TLC (CHCl_3_/MeOH 90:10, *v*/*v*): R*_f_* = 0.35; ${\left[\alpha \right]}_{D}^{20}$ = −11.8 (c 1.0, CH_3_OH); ^1^H-NMR (300 MHz, CDCl_3_) δ 7.74 (d, 2H, *J* = 6.2 Hz, ArH Fmoc), 7.56 (d, 2H, *J* = 6.2 Hz, ArH Fmoc), 7.36 (t, 2H, *J* = 6.2 Hz, ArH Fmoc), 7.29 (t, 2H, *J* = 6.2 Hz, ArH Fmoc), 5.71 (bs, 1H, NHCO), 5.40 (bs, 1H, NHCO), 4.41 (d, 2H, *J* = 6.1 Hz, CH_2_ Fmoc), 4.24–4.36 (m, 1H, C*H*NH), 4.19 (t, 1H, *J* = 6.1 Hz), 3.51–3.72 (m, 2H, C*H*_2_NH), 1.45 (s, 9H, *t*Bu); ^13^C-NMR (75 MHz, CDCl_3_) δ 172.3, 157.6, 157.1, 143.8, 141.4, 127.8, 127.1, 125.0, 120.0, 67.5, 54.8, 47.2, 42.7, 34.0, 28.1. MALDI HRMS: calculated for C_23_H_27_N_2_O_6_^+^ 427.1864, found 427.1881.

### 4.8. Methylation of ***20***. Synthesis of Fmoc-L-Dap(Boc)-OCH_3_ *(****21****)*

The purified amino acid **20** (404.7 mg, 0.95 mmol) was treated with an excess of a 0.66 N solution of diazomethane in dry DCM (5 mL), under magnetic stirring, for 15 min at room temperature. The mixture was evaporated to dryness under reduced pressure conditions (CAUTION: use a fume hood), and the solid residue was co-evaporated twice from small portions of a 2:1 (*v*/*v*) MTB/*n*-pentane mixture, to give pure **21** without need for chromatography. White powder (417.8 mg, quantitative); mp >83 °C (dec.); TLC (CHCl_3_/MeOH 90:10, *v*/*v*): R*_f_* = 0.65. ${\left[\alpha \right]}_{D}^{20}$ = −12.5 (c 1.0, CH_3_OH; Lit. [51]: −10 and −14; ^1^H-NMR (300 MHz, CDCl_3_) δ 7.76 (d, 2H, *J* = 6.2 Hz, ArH Fmoc), 7.57 (d, 2H, *J* = 6.2 Hz, ArH Fmoc), 7.39 (t, 2H, *J* = 6.2 Hz, ArH Fmoc), 7.28 (t, 2H, *J* = 6.2 Hz, ArH Fmoc), 5.32 (bs, 1H, NHCO), 5.11 (bs, 1H, NHCO), 5.40 (bs, 1H, NHCO), 4.30–4.42 (m, 3H, C*H*NH and CH_2_ Fmoc), 4.21 (t, 1H, *J* = 6.1 Hz, CH Fmoc), 3.75 (s, 3H, OCH_3_), 3.50-3.63 (m, 2H, C*H*_2_NH), 1.45 (s, 9H, *t*Bu); ^13^C-NMR (75 MHz, CDCl_3_) δ 171.0, 156.7, 155.4, 143.9, 141.4, 127.7, 127.1, 125.0, 120.0, 80.4, 67.1, 54.2, 52.6, 47.3, 43.2, 28.3; MALDI HRMS: calculated for C_24_H_29_N_2_O_6_^+^ 441.2020, found 441.1998.

### 4.9. Coupling of ***21*** with Fmoc-l-Ala-OH and Fmoc-d-Ala-OH. Synthesis of diastereomers ***23*** and ***24***

DEA (2 mL) was added to a solution of **21** (356.4 mg, 0.81 mmol), in dry DCM (5 mL), under magnetic stirring at room temperature. After 30 min, the volatile was removed under reduced pressure conditions, to afford a solid residue which was dissolved in the minimum volume of EtOAc and precipitated from cold *n*-pentane. The supernatant was separated, and the solid residue was washed with small portions of cold *n*-pentane (5 × 5 mL), then dried under vacuum. The recovered crude product **22** was dissolved in DCM (3 mL) and added to a magnetically stirred mixture of DIEA (0.35 mL; 2.03 mmol), HOBt monohydrate (150 mg; 0.97 mmol), and EDC (160 mg; 0.85 mmol) in DCM (5 mL). After 15 min at room temperature, the appropriate enantiomer of Fmoc-Ala-OH (311.3 mg; 1.0 mmol), dissolved in DCM (3 mL), was added dropwise, and the mixture was allowed to stir overnight at room temperature. The solvent was then evaporated to dryness under reduced pressure conditions, and the residue was solubilized in EtOAc (10 mL), washed once with 5% aqueous NaHCO_3_ (5 mL), once with 5% aqueous KHSO_4_ (5 mL), and once with brine (5 mL). The organic layer was dried over Na_2_SO_4_, paper-filtered, and evaporated to dryness to yield the expected dipeptides **23** and **24**, both as a white powder. The pair of crude diastereomers was directly analyzed by RP U-HPLC/MS, without further pre-injection purification (Supplementary Materials).

## 5. Conclusions

In summary, a synthetic route is proposed for the multi-step preparation of orthogonal di-protected l-Dap methyl esters. In the proposed procedure, commercial *N*^α^-Fmoc-*O*-*tert*-butyl-d-serine is initially transformed into the corresponding serinal. This compound is subjected to a Ti(IV)-assisted reductive amination with amines and arylsulfonamides, to give 2,3-diaminopropanols which are in turn oxidized by the TCCA/TEMPO system. The obtained carboxylic acids are finally methylated, to yield the targeted esters in 68%–73% overall yields. Reductive amination allows also the preparation of *N*-methylated and *N*-ethylated 2,3-diaminopropanol intermediates as the most likely precursors of *N*-alkylated l-Dap scaffolds. Chromatographic purifications are minimized through the entire procedure. The total preservation of chirality enhanced the synthetic utility of the method, which can successfully be used to prepare protected l-Dap building blocks for a broad application in the synthesis of biologically active peptides.

## Figures and Tables

**Table 1:** Reductive amination of **3**: synthesis of the 2,3-diaminopropanol intermediates.

Reagent	Product	2,3-Diaminopropanol Structure	Yield (%) ^1^
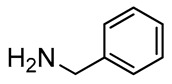	**4**	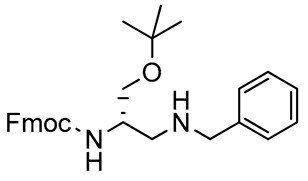	92
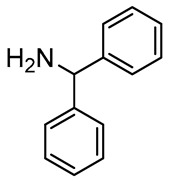	**5**	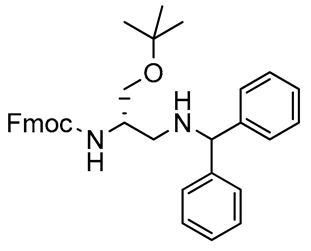	90
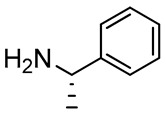	**6**	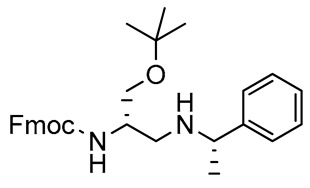	90
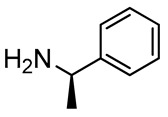	**7**	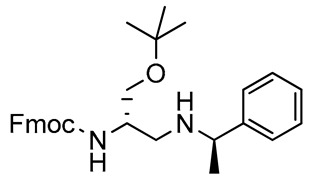	91
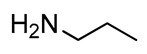	**8**	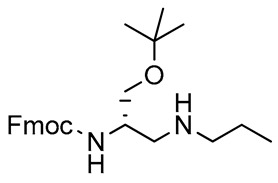	89
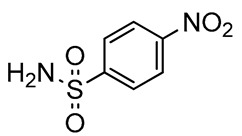	**9**	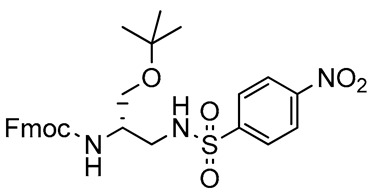	82
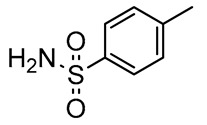	**10**	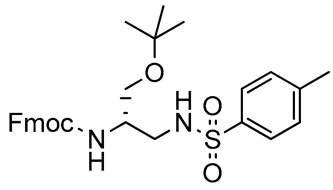	85

^1^ Yields in final products obtained after chromatography.

## Supplementary Materials

The following are available online at https://www.mdpi.com/1420-3049/25/6/1313/s1: Figure S1: ^1^H spectrum of **2**; Figure S2: ^13^C spectrum of **2**; Figure S3: ^1^H spectrum of **3**; Figure S4: ^13^C spectrum of **3**; Figure S5: ^1^H spectrum of **4**; Figure S6: ^13^C spectrum of **4**; Figure S7: ^1^H spectrum of **5**; Figure S8: ^13^C spectrum of **5**; Figure S9: ^1^H spectrum of **6**; Figure S10: ^13^C spectrum of **6**; Figure S11: ^1^H spectrum of **7**; Figure S12: ^13^C spectrum of **7**; Figure S13: ^1^H spectrum of **8**; Figure S14: ^13^C spectrum of **8**; Figure S15: ^1^H spectrum of **9**; Figure S16: ^13^C spectrum of **9**; Figure S17: ^1^H spectrum of **10**; Figure S18: ^13^C spectrum of **10**; Figure S19: ^1^H spectrum of **11**; Figure S20: ^13^C spectrum of **11**; Figure S21: ^1^H spectrum of **12**; Figure S22: ^13^C spectrum of **12**; Figure S23: ^1^H spectrum of **13**; Figure S24: ^13^C spectrum of **13**; Figure S25: ^1^H spectrum of **20**; Figure S26: ^13^C spectrum of **20**; Figure S27: ^1^H spectrum of **21**; Figure S28: ^13^C spectrum of **21**; Figures S29–S36: ESI(+) MS spectra of **4**–**11**; Figure S37: RP U-HPLC analysis of crude **23** and crude **24**; Figures S38 and S39: ESI(+) MS spectra of **23** and **24**.

## Abbreviations

TEMPO (2:2,6,6-tetramethylpiperidin-1-yl)oxyl, TCCA (trichloroisocyanuric acid); DEA (*N*,*N*-diethylamine), DIEA (*N*,*N-*diisopropylethylamine); HOBt (1-hydroxybenzotriazole); DIC (*N*,*N*′-diisopropylcarbodiimide); EDC (1-(3-dimethylaminopropyl)-3-ethylcarbodiimide hydrochloride); Boc_2_O (di-*tert*-butyl-dicarbonate); Fmoc-OSu (*N*-(9-fluorenylmethoxycarbonyloxy)succinimide); TFA (trifluoroacetic acid); TFE (trifluoroethanol); THF (tetrahydrofuran); DCM (dichloromethane); AcOEt (ethyl acetate); CH_3_CN (acetonitrile); MTB (methyl-*tert*-butyl ether); TLC (thin-layer chromatography); FCC (flash column chromatography); RP U-HPLC/MS (reversed-phase ultrafast high-pressure liquid chromatography/mass spectrometry); HRMS (high-resolution mass spectrometry); ESI(+) MS (positive-mode electro spray ionization mass spectrometry); TOF (time of flight); MS/MS (tandem mass spectrometry); TOCSY (total correlation spectroscopy).
