# Supplementary material for: 2,3-Diaminopropanols Obtained from d-Serine as Intermediates in the Synthesis of Protected 2,3-l-Diaminopropanoic Acid (l-Dap) Methyl Esters
Source: Molecules. 2020 Mar 13;25(6):1313. doi: 10.3390/molecules25061313 (PMC7145313; doi:10.3390/molecules25061313)

2,3-Diaminopropanols obtained from D-serine as intermediates in the synthesis of protected 2,3-L-diaminopropanoic acid (L-Dap) methyl esters

Andrea Temperini, Donatella Aiello, Fabio Mazzotti, Constantinos M. Athanassopoulos, Pierantonio De Luca and Carlo Siciliano *

Supplementary Materials

Page 2. Figure S1: ^{1}H NMR spectrum of **2**; Figure S2: ^{13}C NMR spectrum of **2**.

Page 3. Figure S3: ^{1}H NMR spectrum of **3**; Figure S4: ^{13}C NMR spectrum of **3**.

Page 4. Figure S5: ^{1}H NMR spectrum of **4**; Figure S6: ^{13}C NMR spectrum of **4**.

Page 5. Figure S7: ^{1}H NMR spectrum of **5**; Figure S8: ^{13}C NMR spectrum of **5**.

Page 6. Figure S9: ^{1}H NMR spectrum of **6**; Figure S10: ^{13}C NMR spectrum of **6**.

Page 7. Figure S11: ^{1}H NMR spectrum of **7**; Figure S12: ^{13}C NMR spectrum of **7**.

Page 8. Figure S13: ^{1}H NMR spectrum of **8**; Figure S14: ^{13}C NMR spectrum of **8**.

Page 9. Figure S15: ^{1}H NMR spectrum of **9**; Figure S16: ^{13}C NMR spectrum of **9**.

Page 10. Figure S17: ^{1}H NMR spectrum of **10**; Figure S18: ^{13}C NMR spectrum of **10**.

Page 11. Figure S19: ^{1}H NMR spectrum of **11**; Figure S20: ^{13}C NMR spectrum of **11**.

Page 12. Figure S21: ^{1}H NMR spectrum of **12**; Figure S22: ^{13}C NMR spectrum of **12**.

Page 13. Figure S23: ^{1}H NMR spectrum of **13**; Figure S24: ^{13}C NMR spectrum of **13**.

Page 14. Figure S25: ^{1}H NMR spectrum of **20**; Figure S26: ^{13}C NMR spectrum of **20**.

Page 15. Figure S27: ^{1}H NMR spectrum of **21**; Figure S28: ^{13}C NMR spectrum of **21**.

Page 16. Figures S29-S30: ESI(+) MS spectra of **4** and **5**.

Page 17. Figures S31-S32: ESI(+) MS spectra of **6** and **7**.

Page 18. Figures S33-S34: ESI(+) MS spectra of **8** and **9**.

Page 19. Figures S35-S36: ESI(+) MS spectra of **10** and **11**.

Page 20. Figure S37: RP U-HPLC/MS analysis of crude **23** and crude **24**.

Page 21. Figures S38-S39: ESI(+) MS spectra of **23** and **24**.

Figure S1: ^{1}H NMR spectrum of **2**

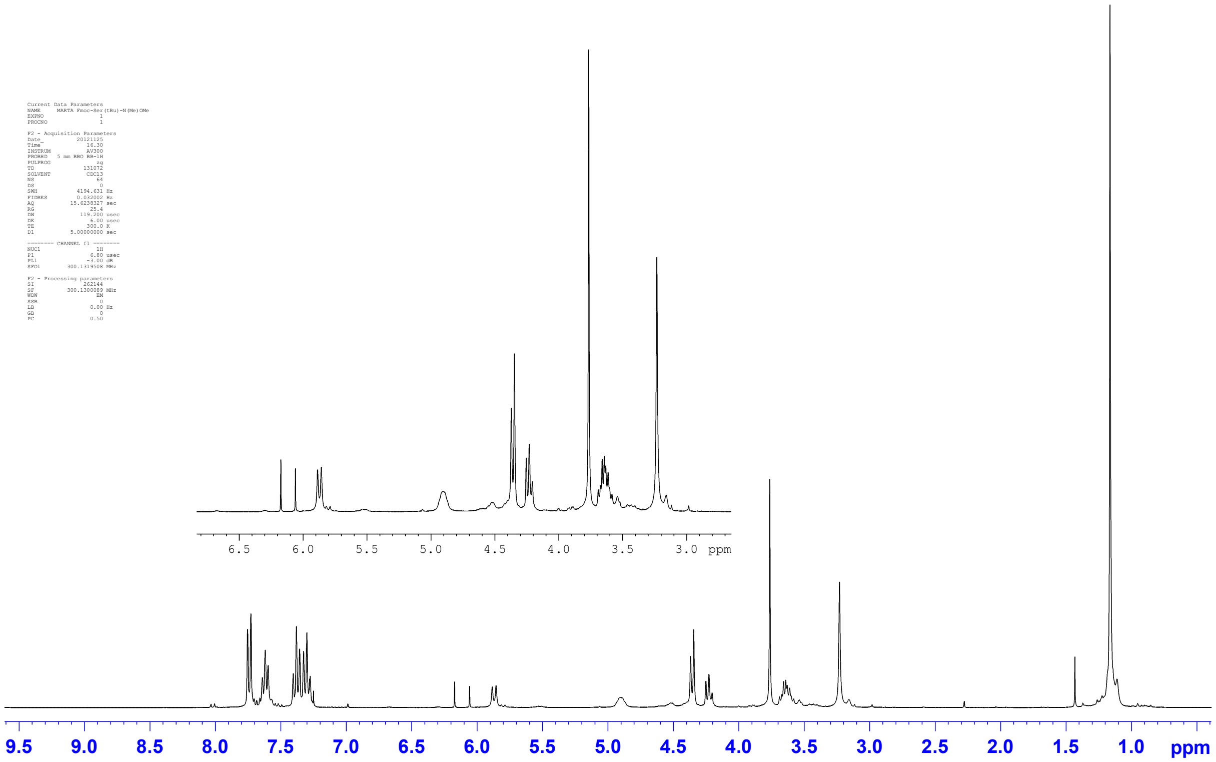

Figure S2: ^{13}C NMR spectrum of **2**

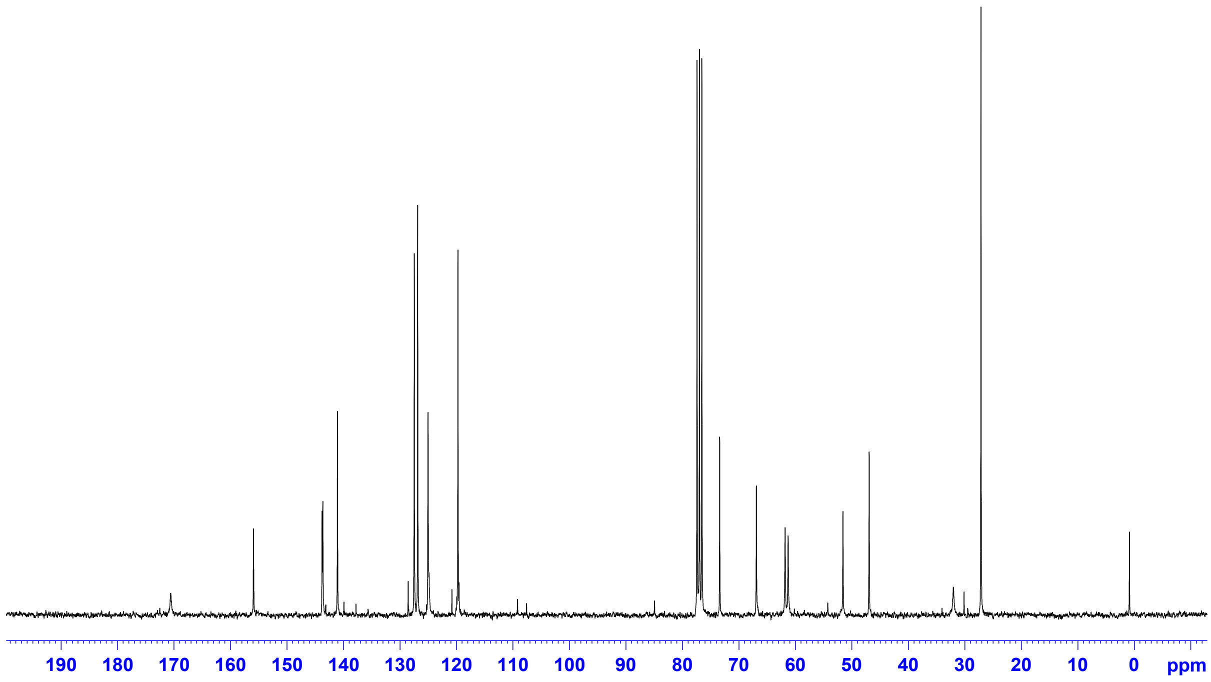

Figure S3: ^{1}H NMR spectrum of **3**

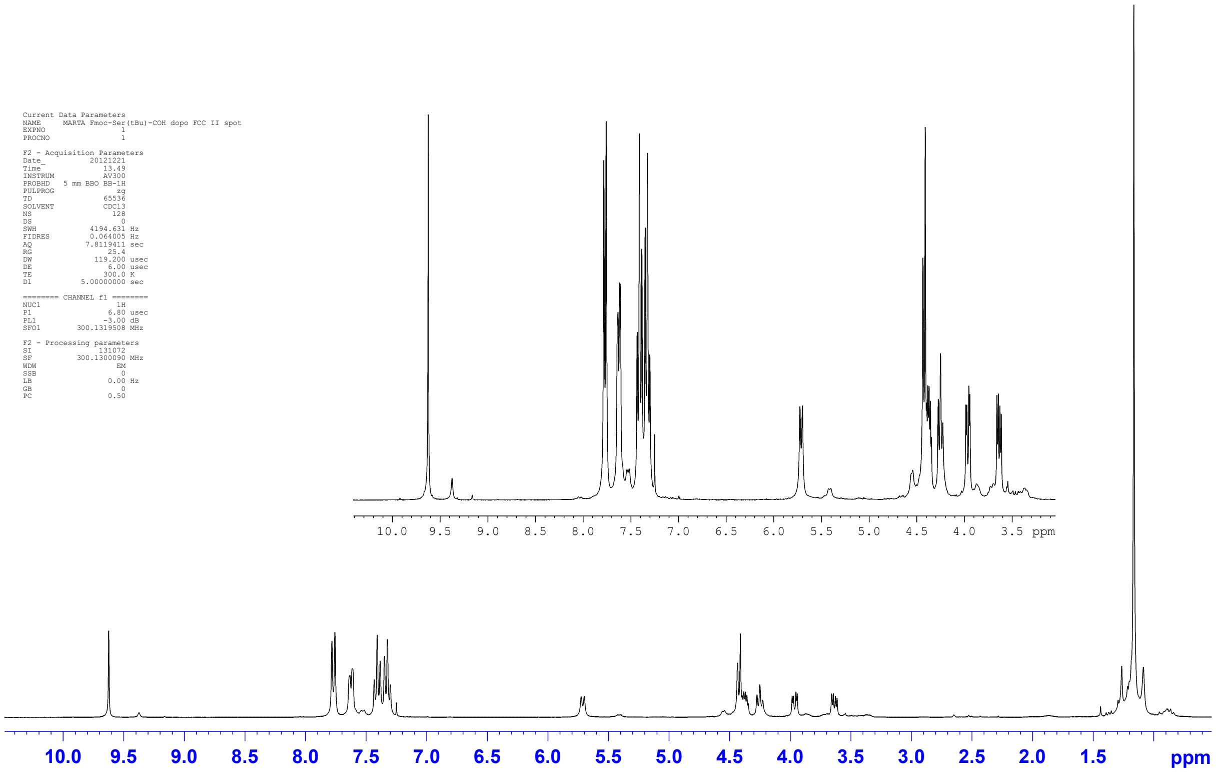

Figure S4: ^{13}C NMR spectrum of **3**

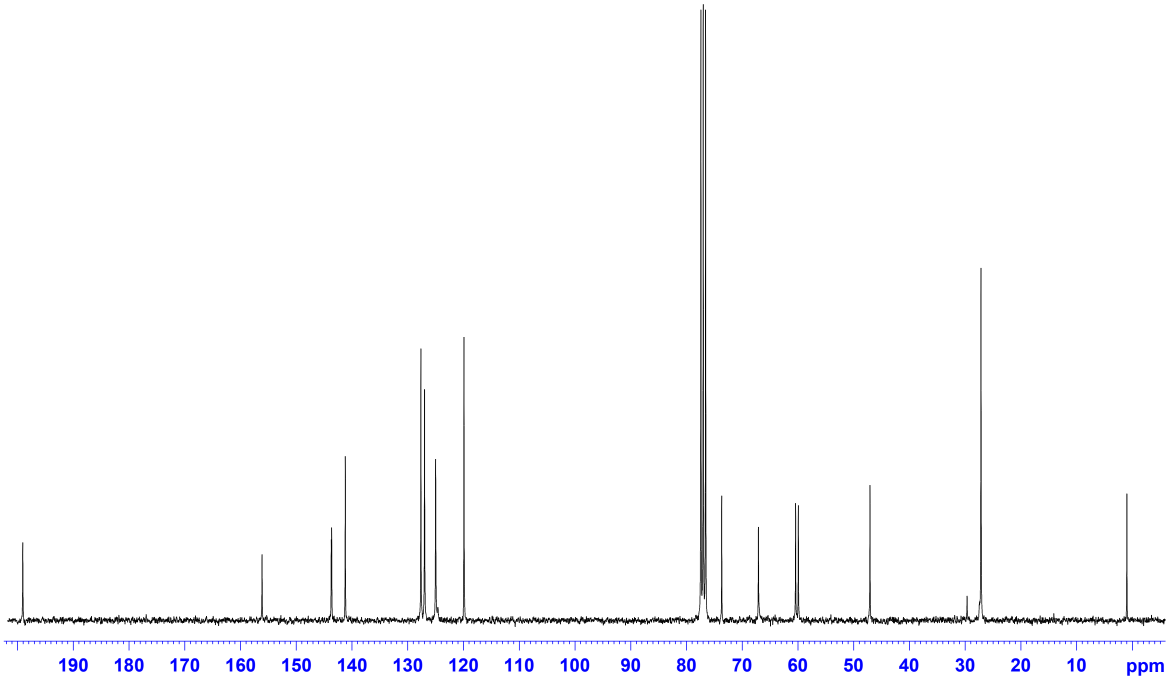

Figure S5: ^{1}H NMR spectrum of **4**

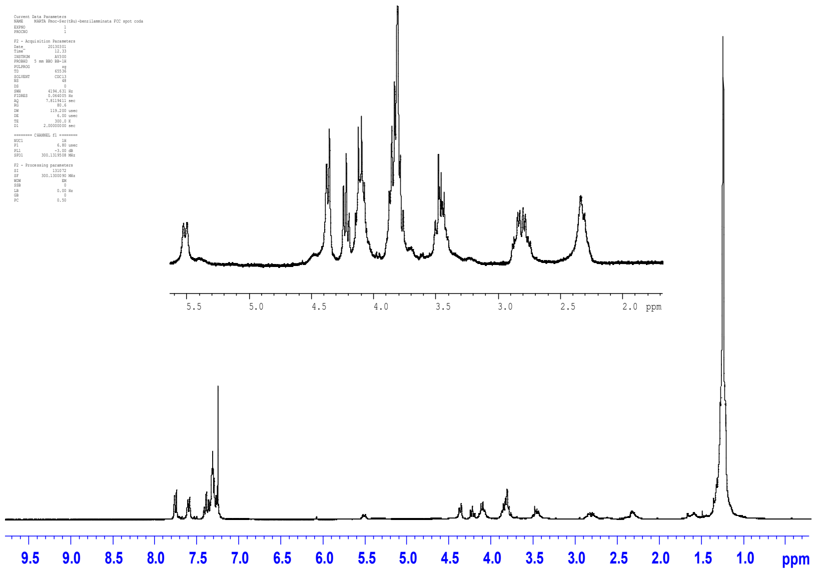

Figure S6: ^{13}C NMR spectrum of **4**

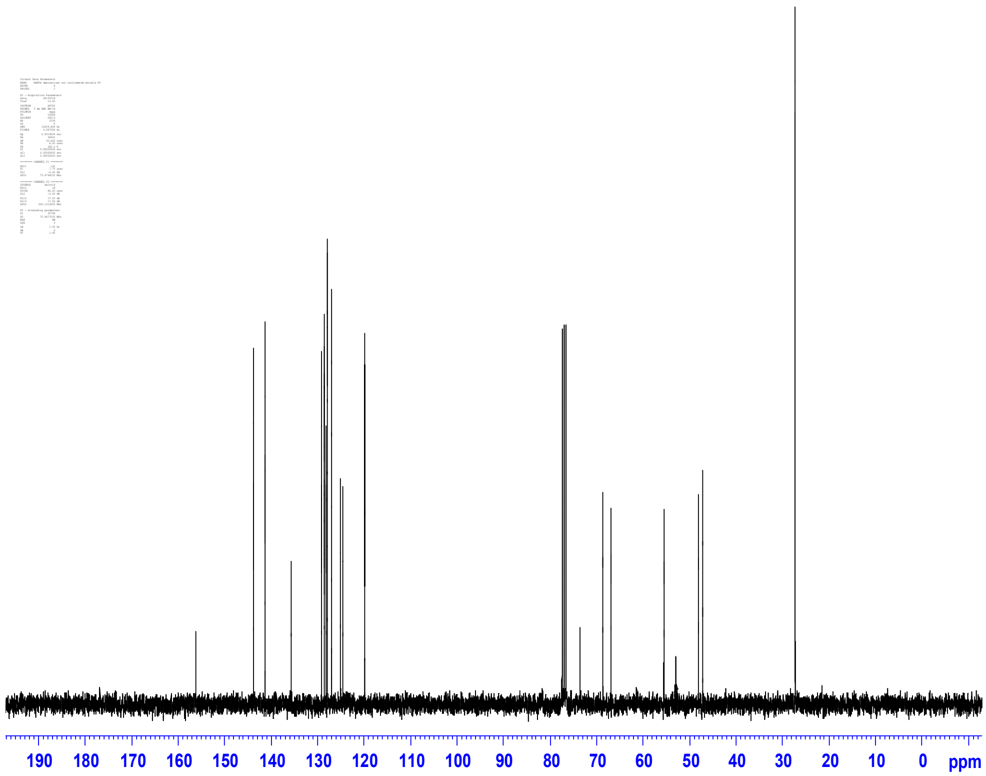

Figure S7: ^{1}H NMR spectrum of **5**

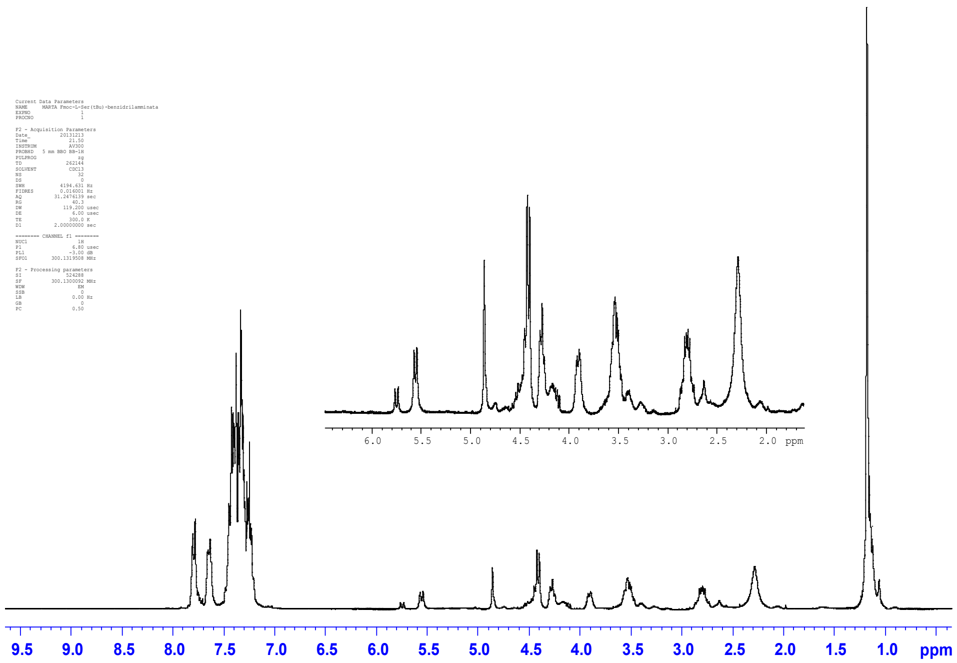

Figure S8: ^{13}C NMR spectrum of **5**

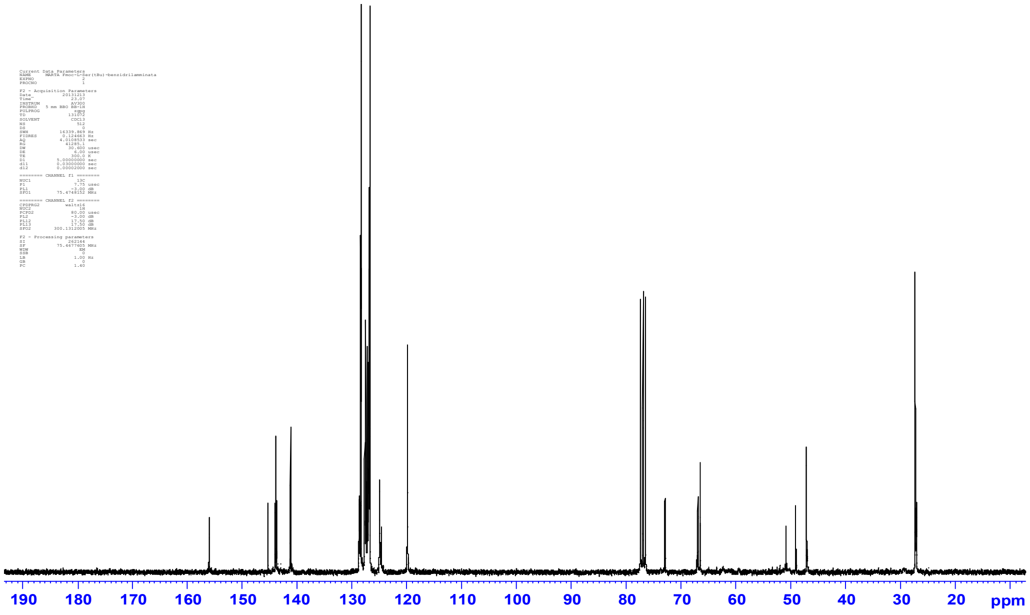

Figure S9: ^{1}H NMR spectrum of **6**

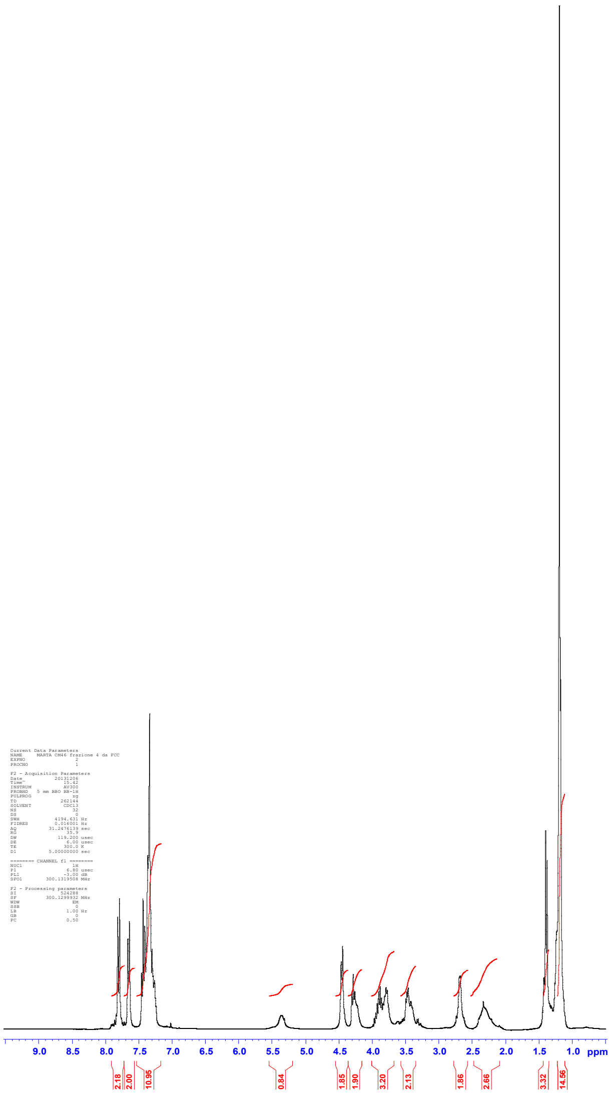

Figure S10: ^{13}C NMR spectrum of **6**

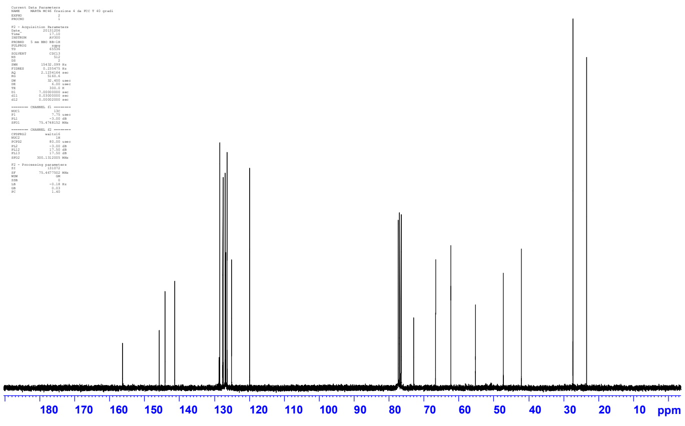

Figure S11: ^{1}H NMR spectrum of **7**

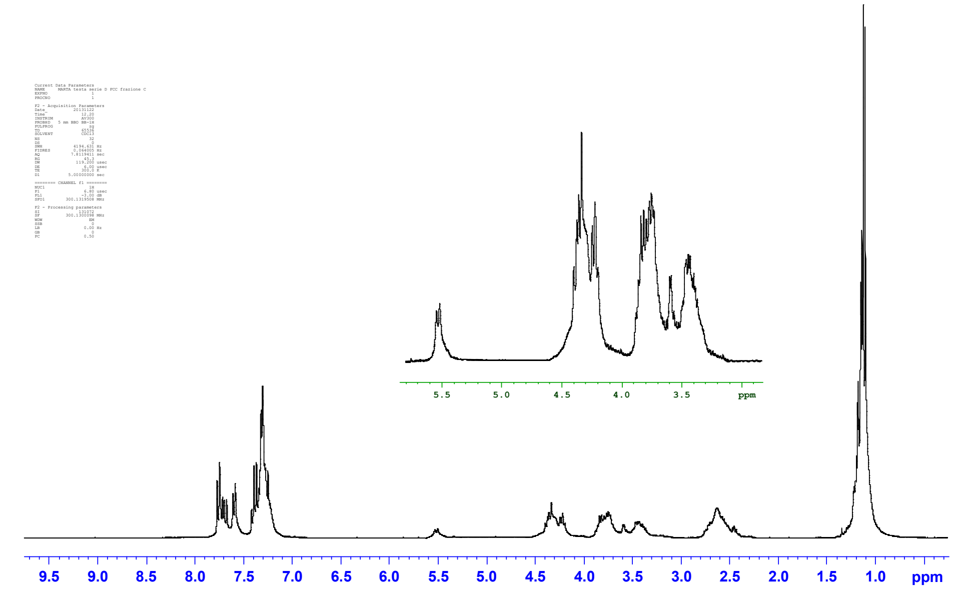

Figure S12: ^{13}C NMR spectrum of **7**

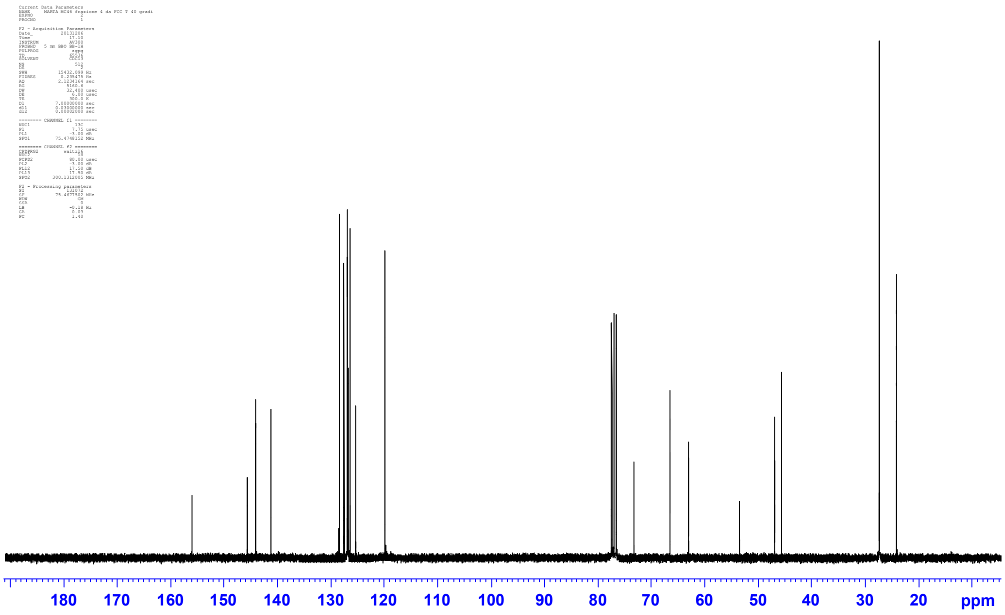

Figure S13: ^{1}H NMR spectrum of **8**

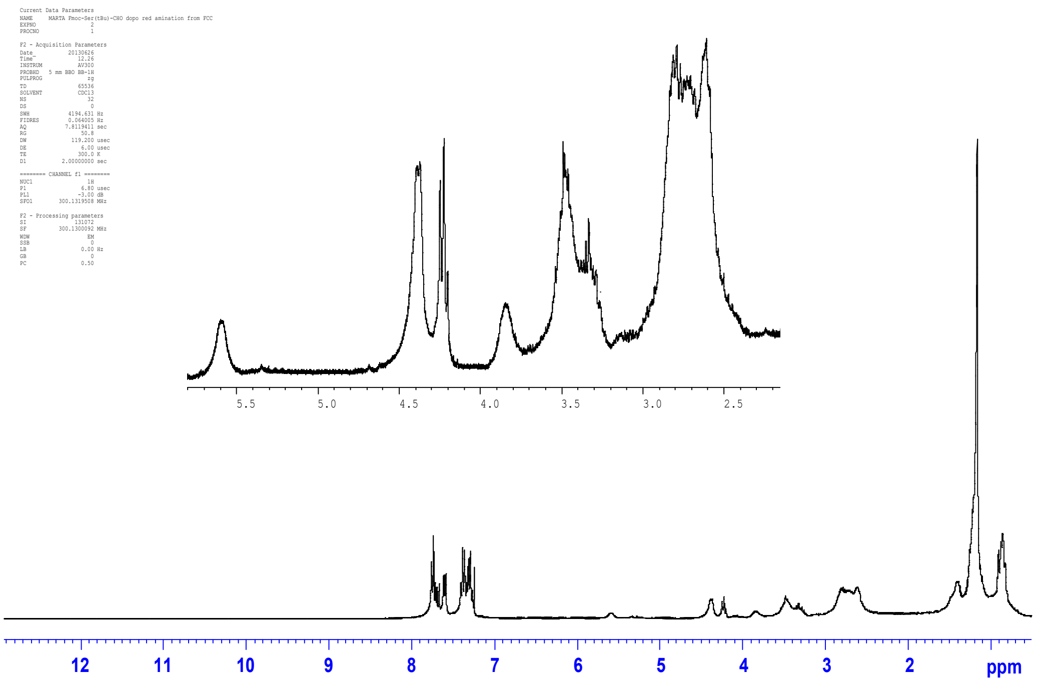

Figure S14: ^{13}C NMR spectrum of **8**

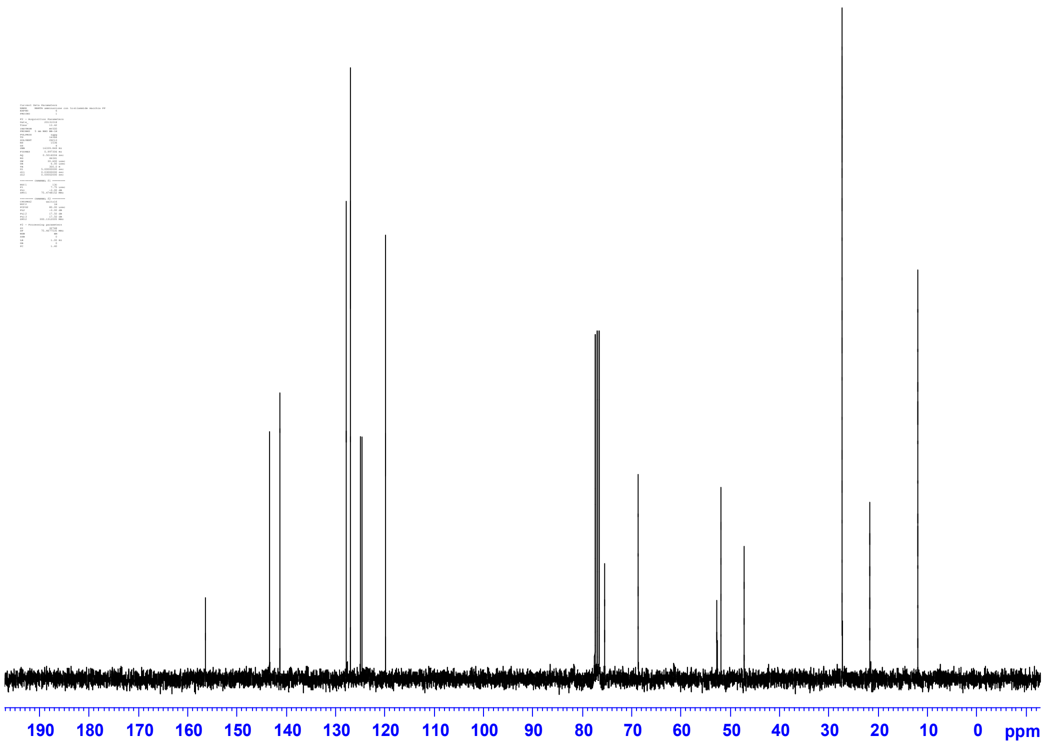

Figure S15: ^{1}H NMR spectrum of **9**

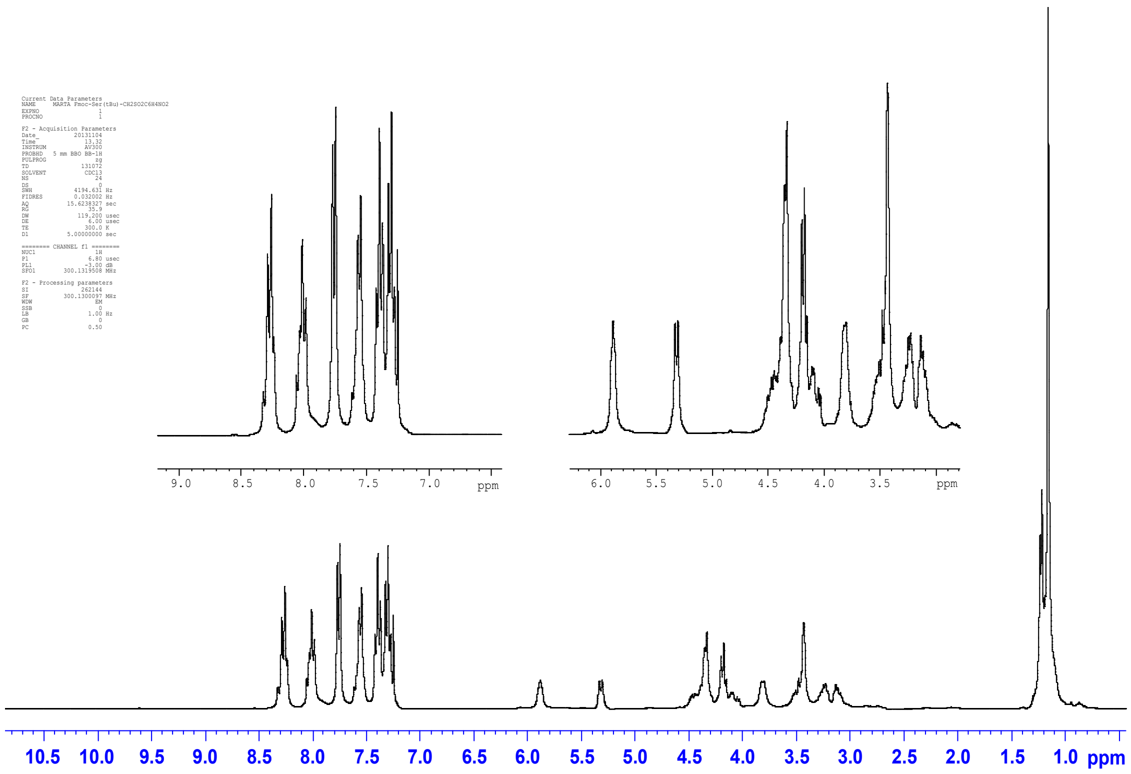

Figure S16: ^{13}C NMR spectrum of **9**

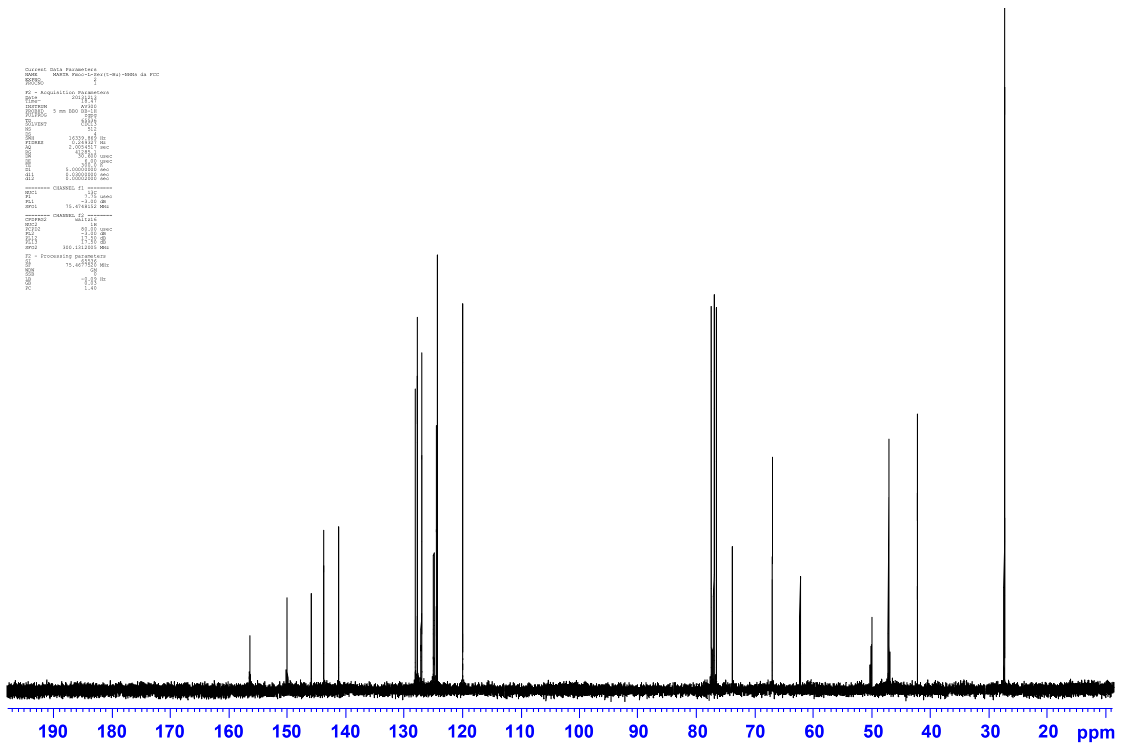

Figure S17: ^{1}H NMR spectrum of **10**

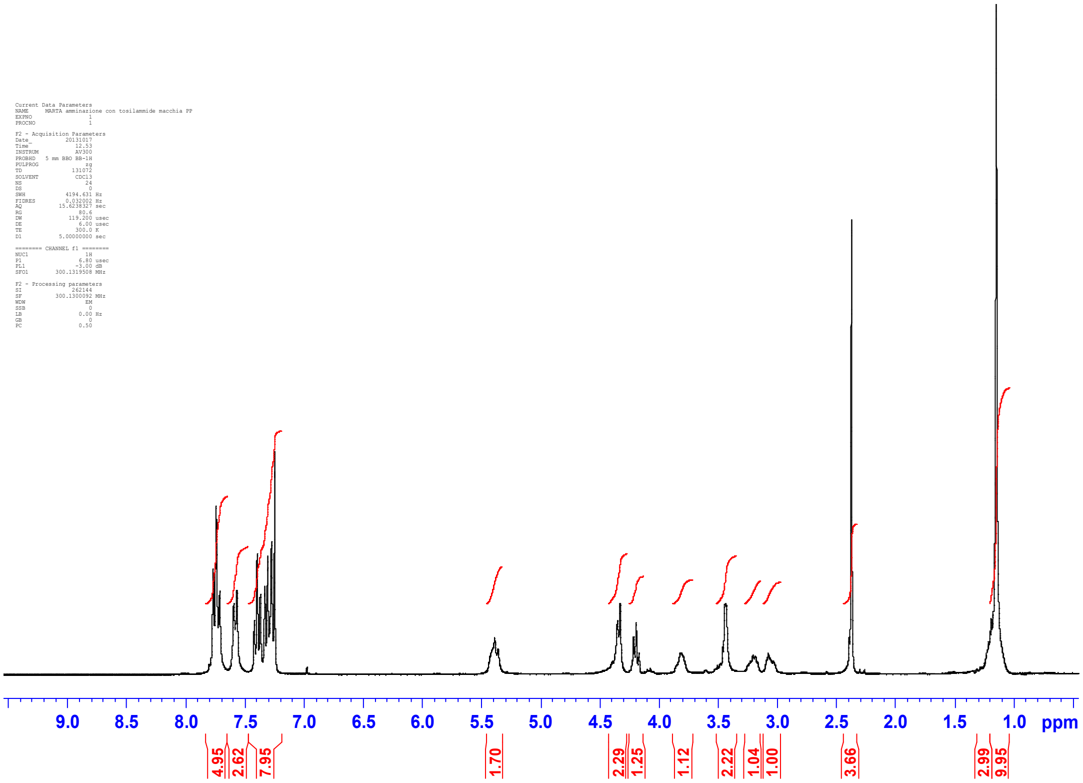

Figure S18: ^{13}C NMR spectrum of **10**

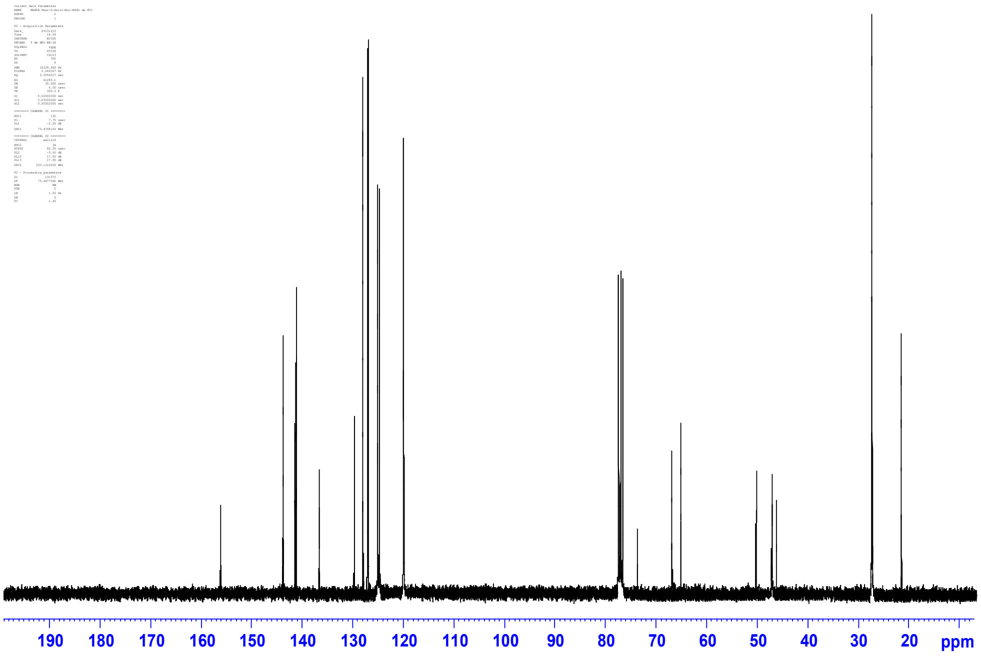

Figure S19: ^{1}H NMR spectrum of **11**

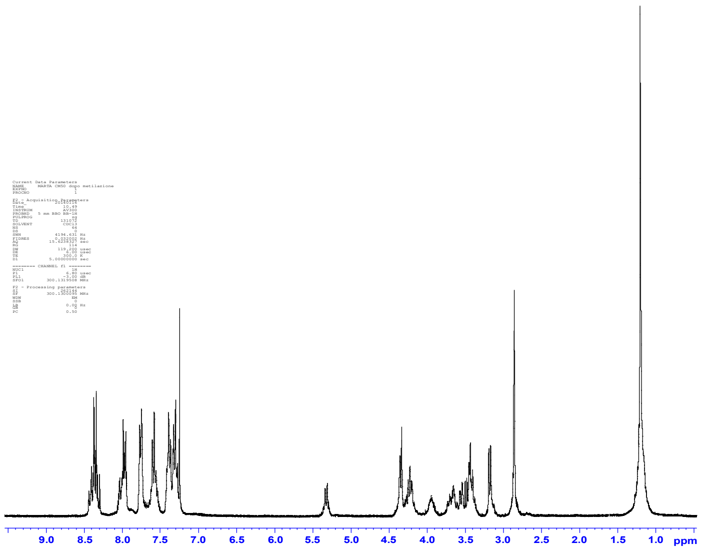

Figure S20: ^{13}C NMR spectrum of **11**

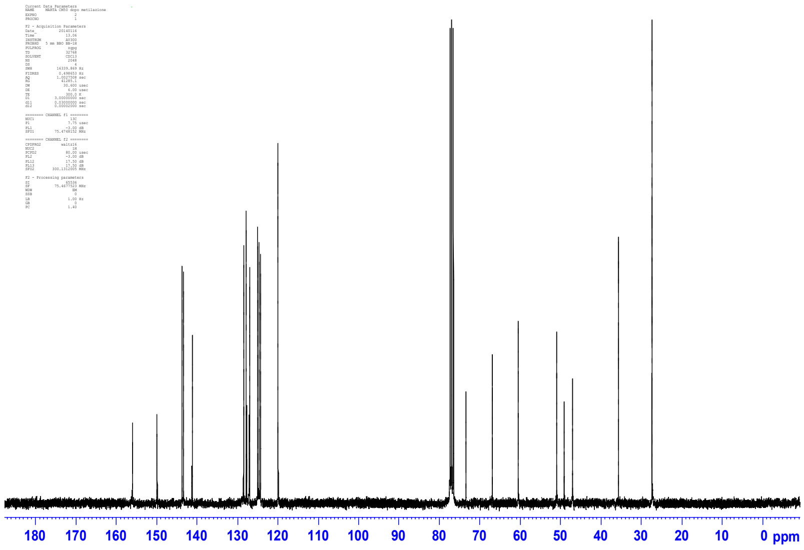

Figure S21: ^{1}H NMR spectrum of **12**

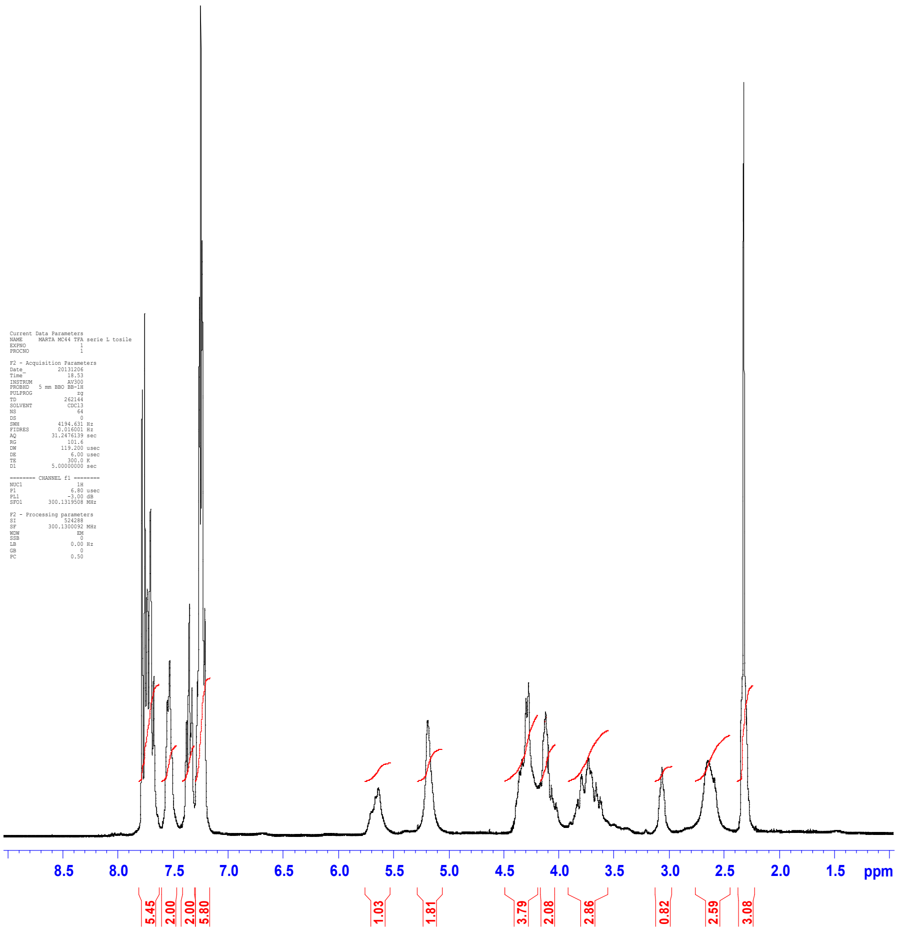

Figure S22: ^{13}C NMR spectrum of **12**

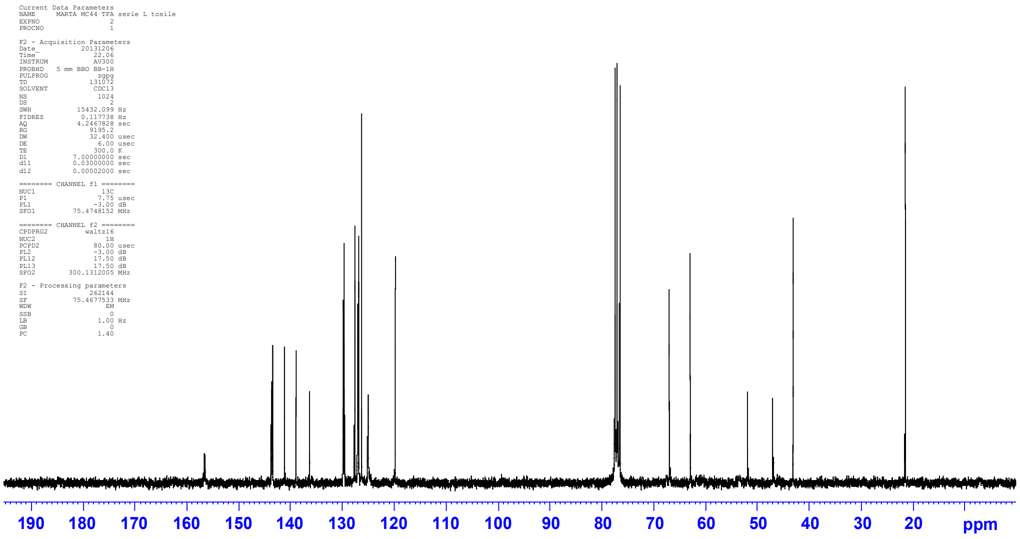

Figure S23: ^{1}H NMR spectrum of **13**

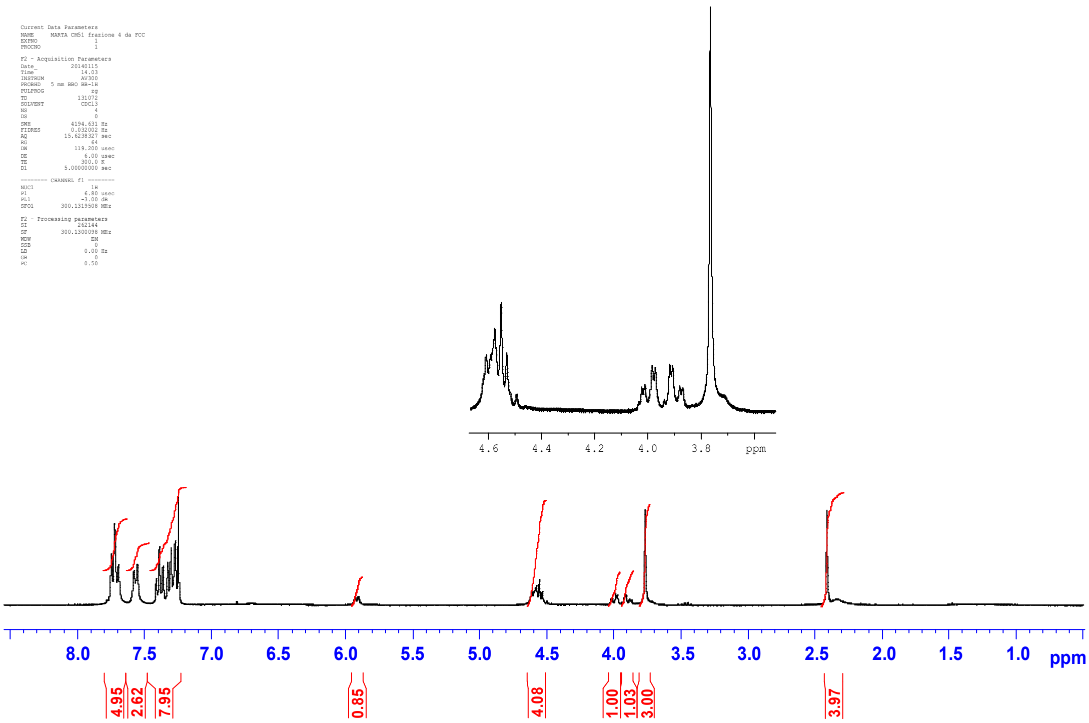

Figure S24: ^{13}C NMR spectrum of **13**

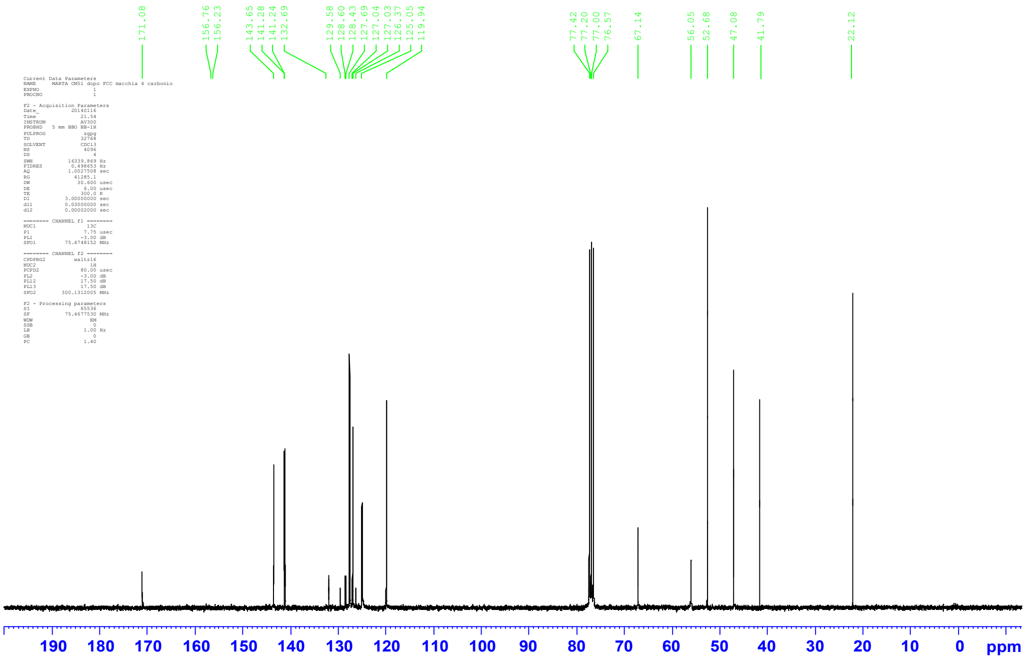

Figure S25: ^{1}H NMR spectrum of **20**

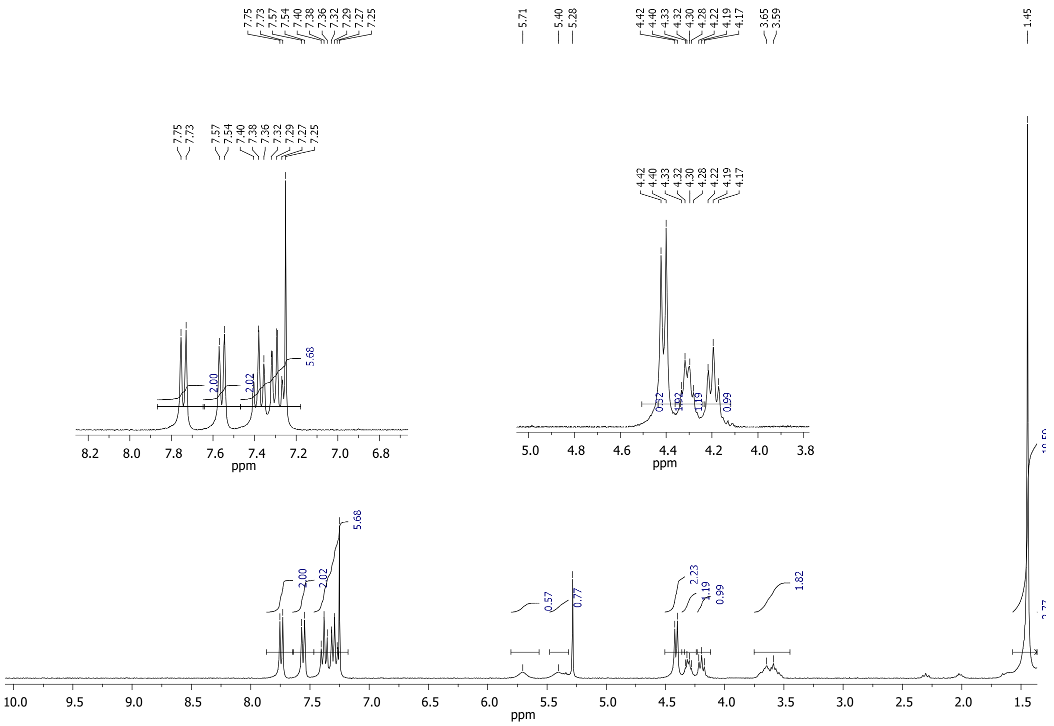

Figure S26: ^{13}C NMR spectrum of **20**

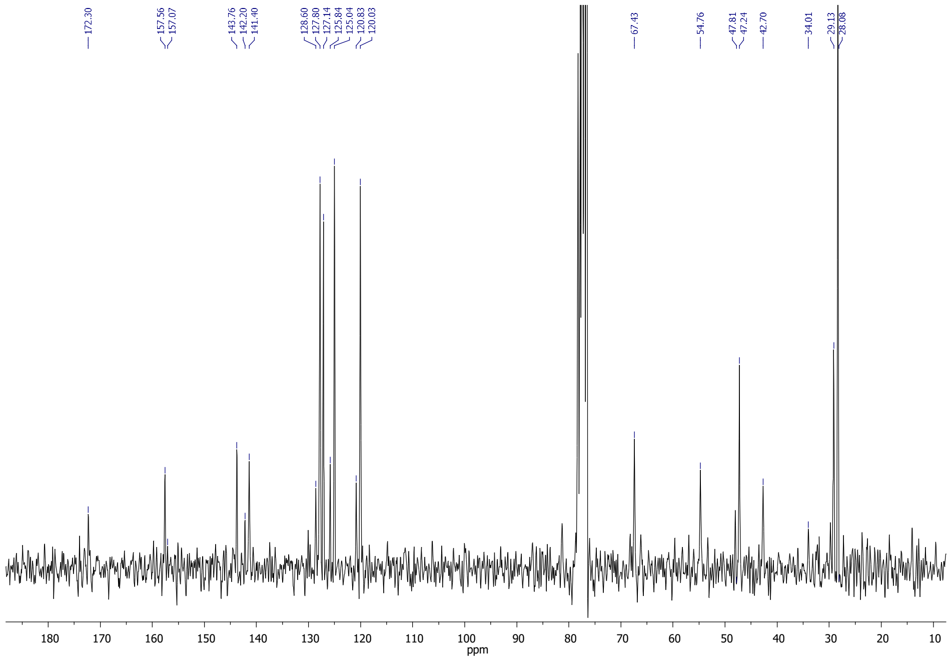

Figure S27: ^{1}H NMR spectrum of **21**

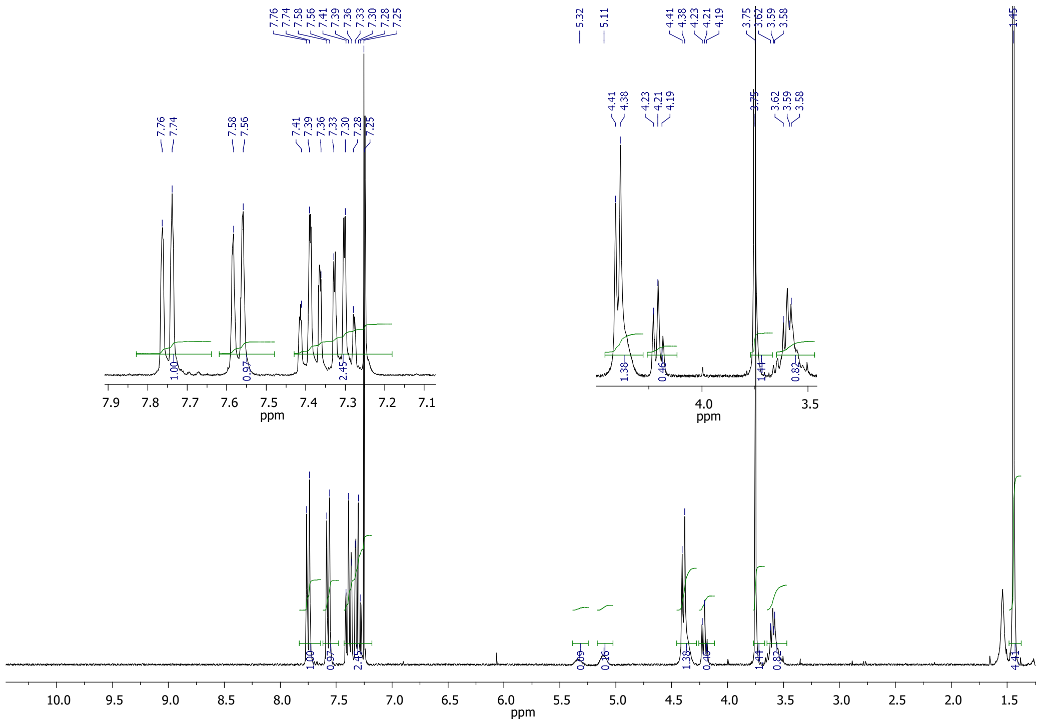

Figure S28: ^{13}C NMR spectrum of **21**

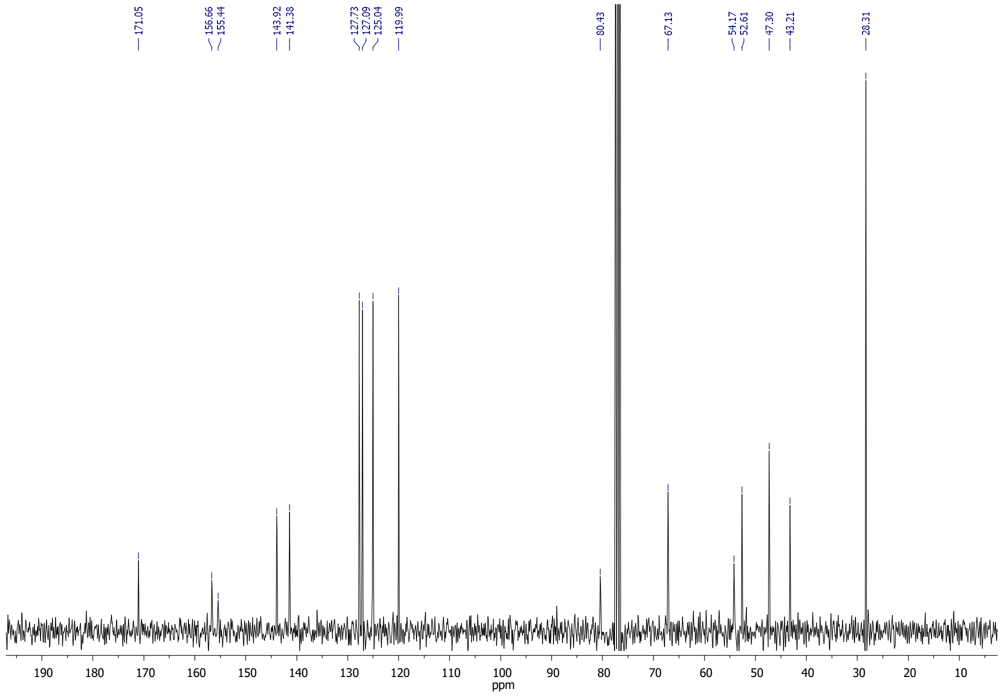

Figure S29: ESI(+) MS spectrum of **4**

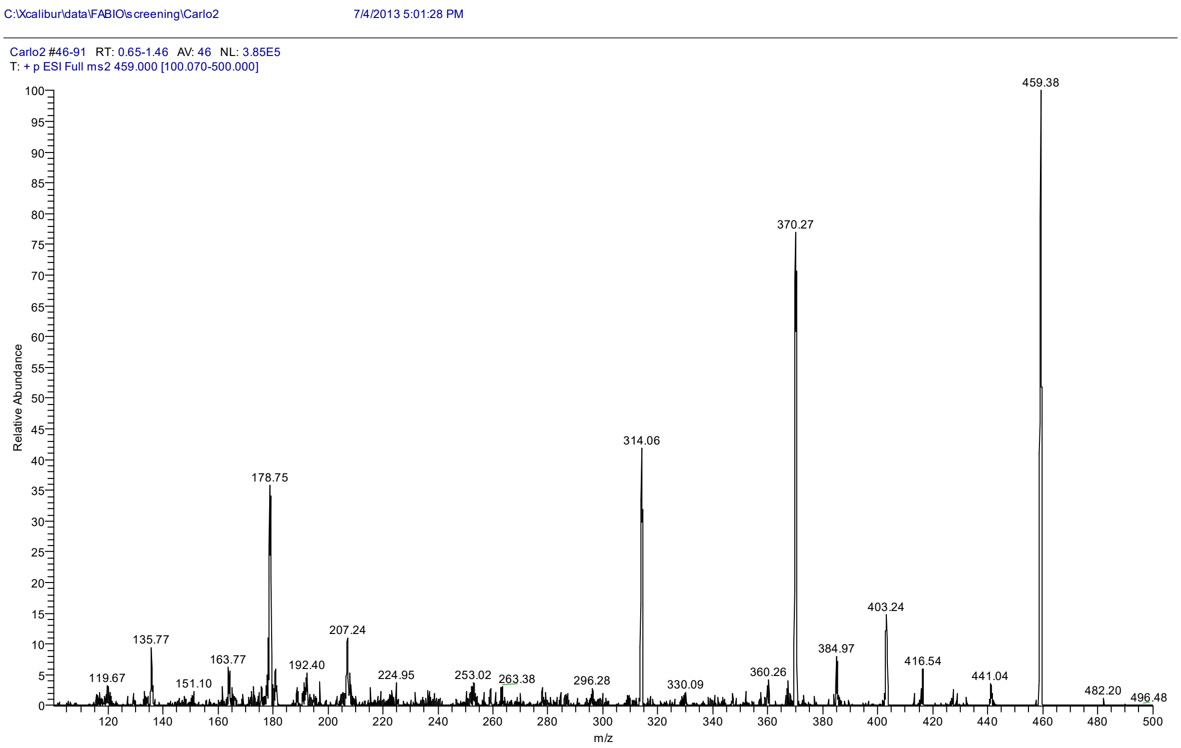

Figure S30: ESI(+) MS spectrum of **5**

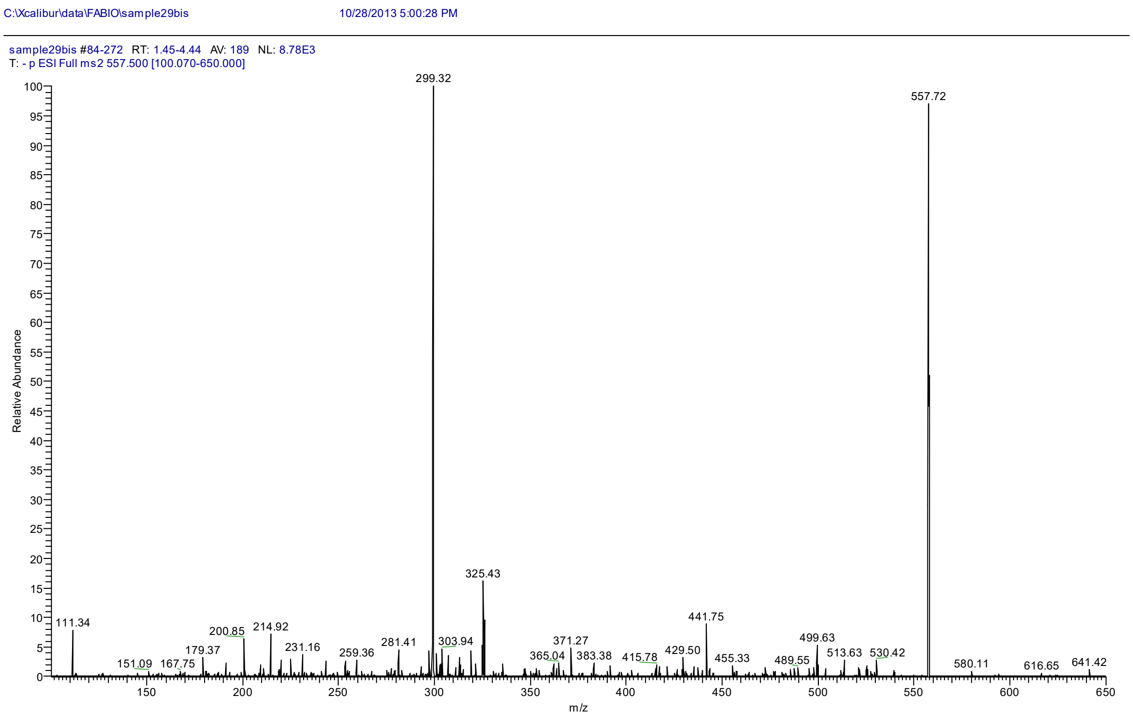

Figure S31: ESI(+) MS spectrum of **6**

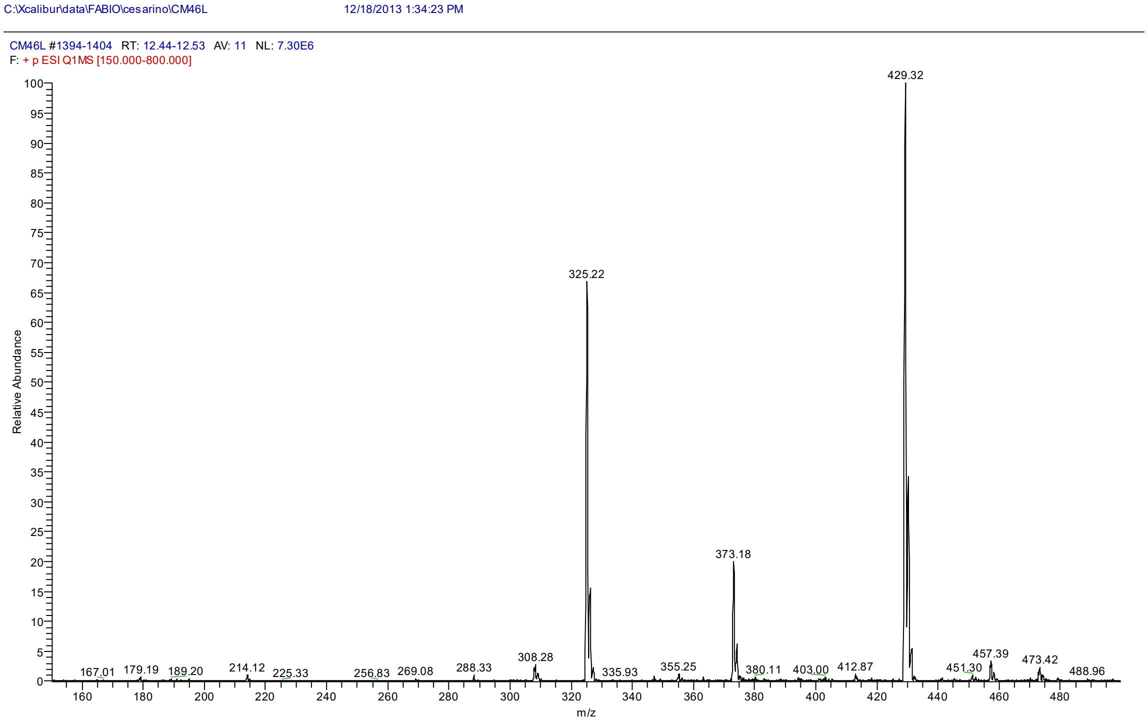

Figure S32: ESI(+) MS spectrum of **7**

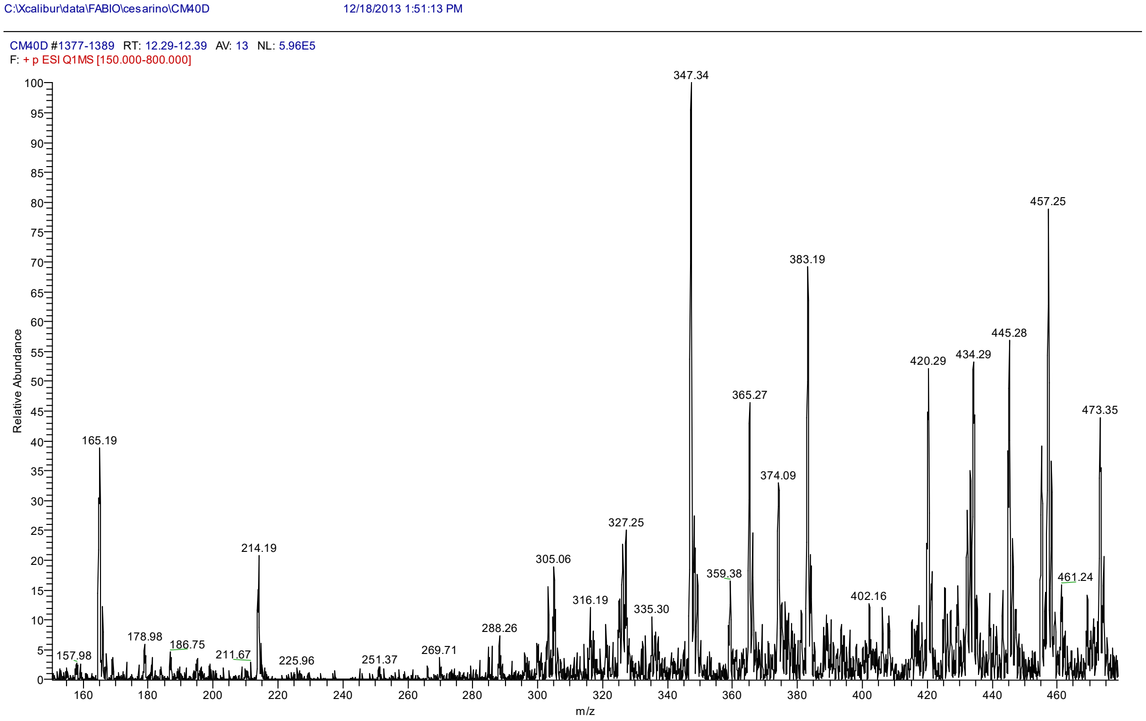

Figure S33: ESI(+) MS spectrum of **8**

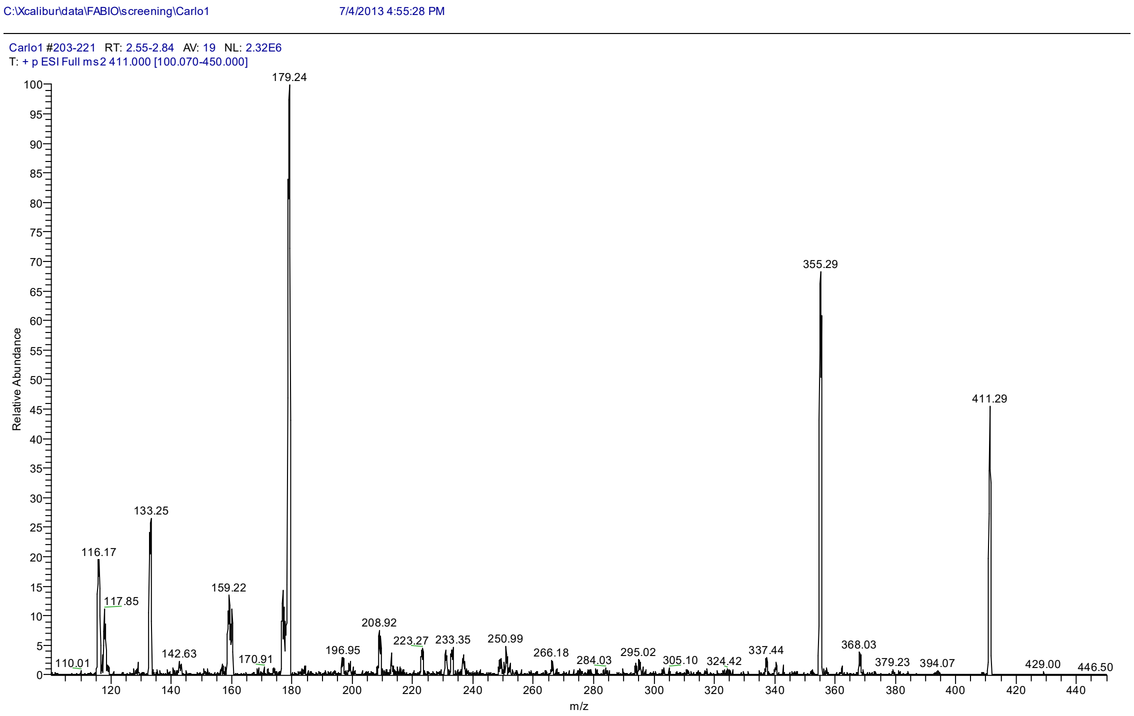

Figure S34: ESI(+) MS spectrum of **9**

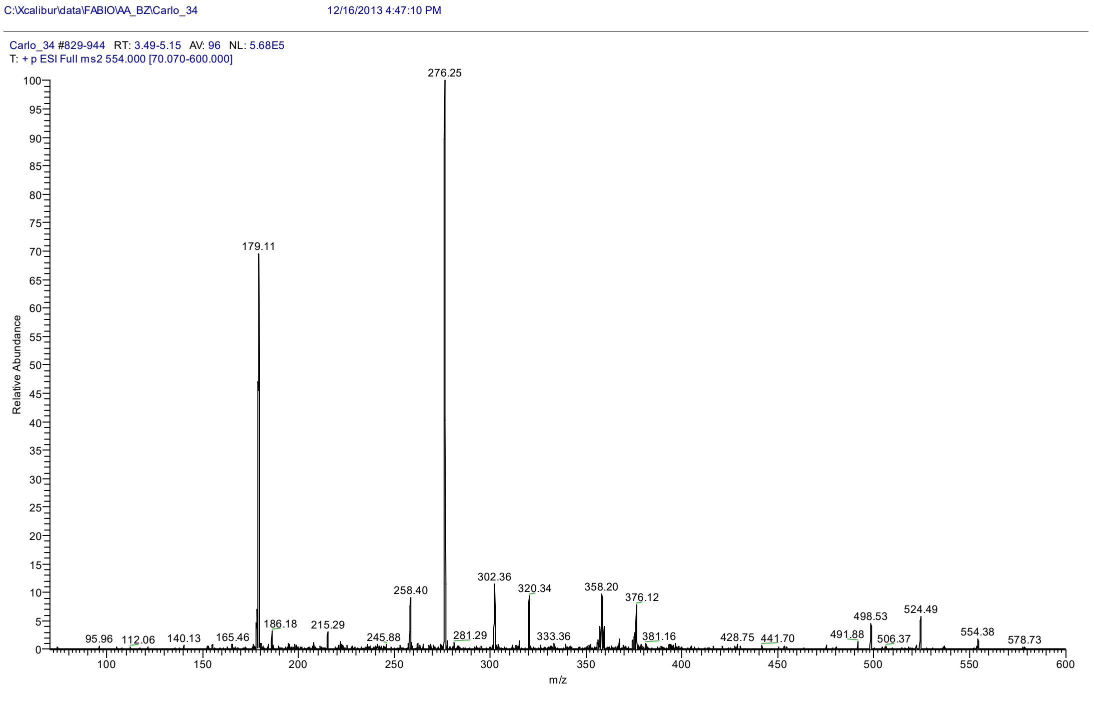

Figure S35: ESI(+) MS spectrum of **10**

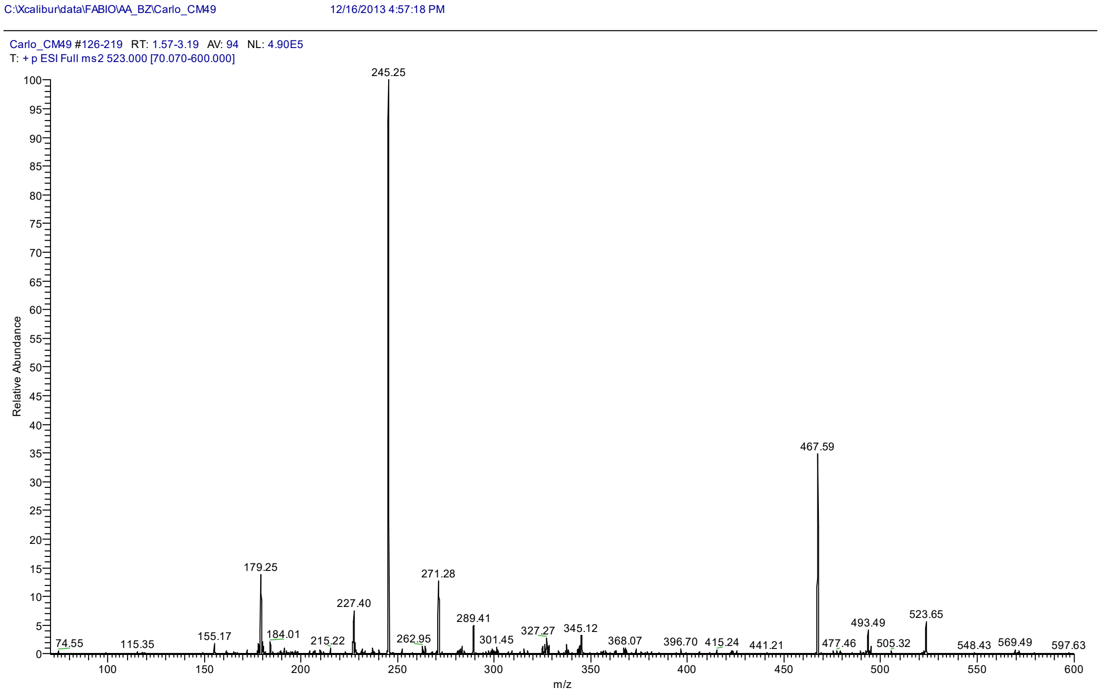

Figure S36: ESI(+) MS spectrum of **11**

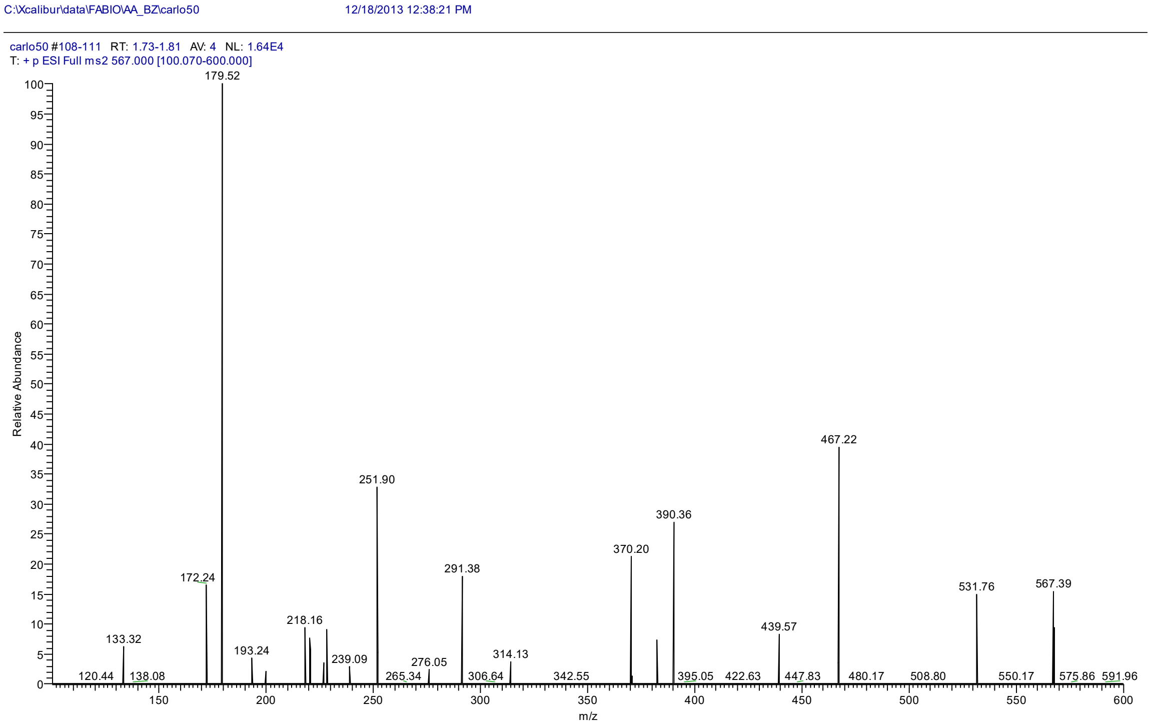

Figure S37: RP U-HPLC/MS analysis of **23** and **24**

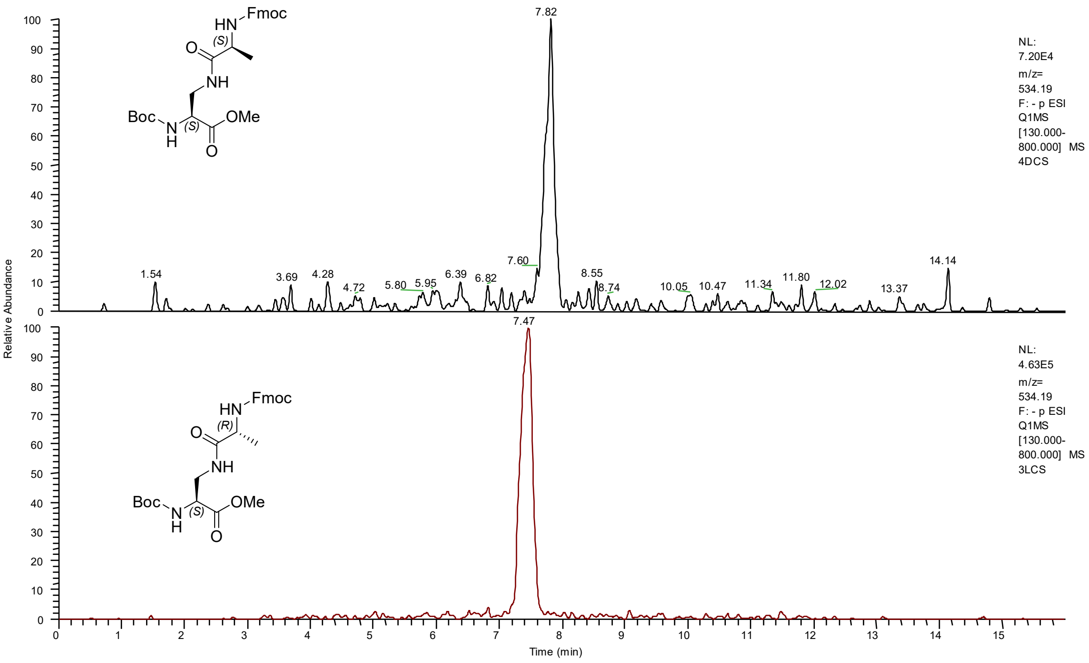

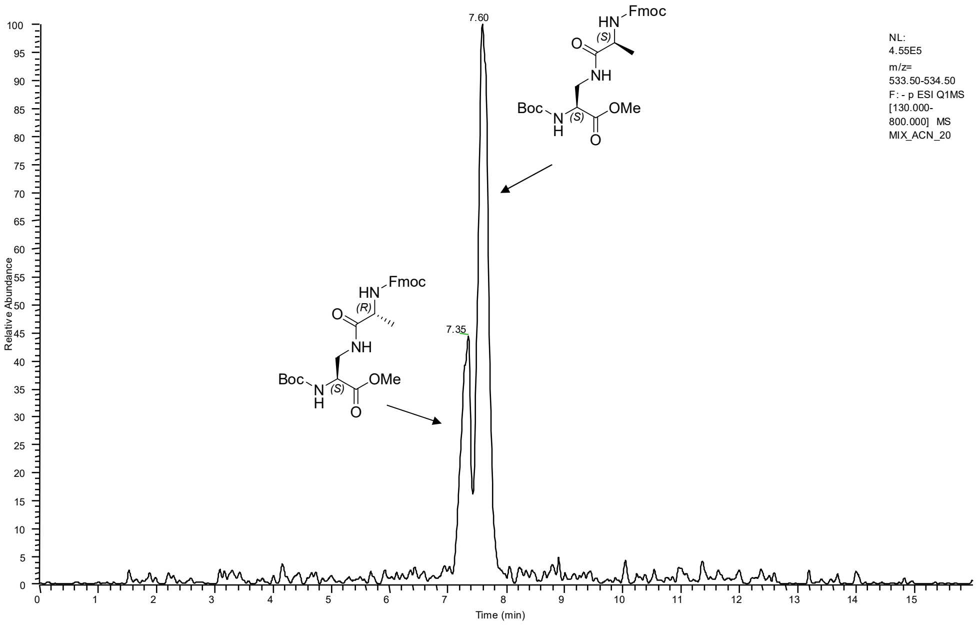

Figure S38: ESI(+) MS spectrum of **23**

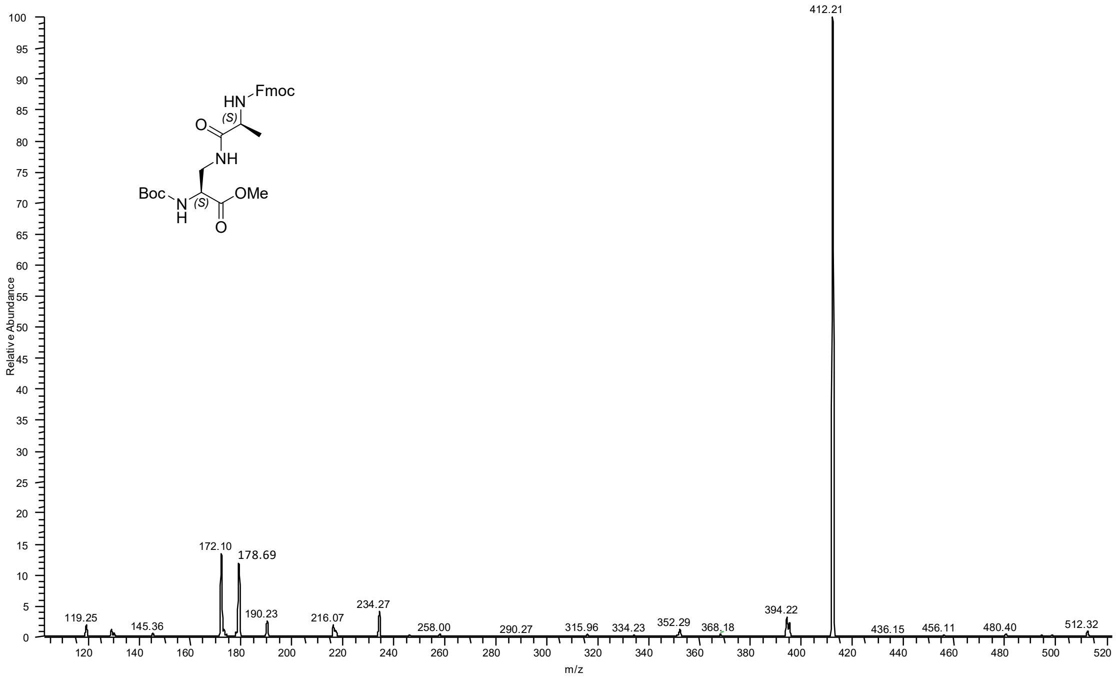

Figure S39: ESI(+) MS spectrum of **24**

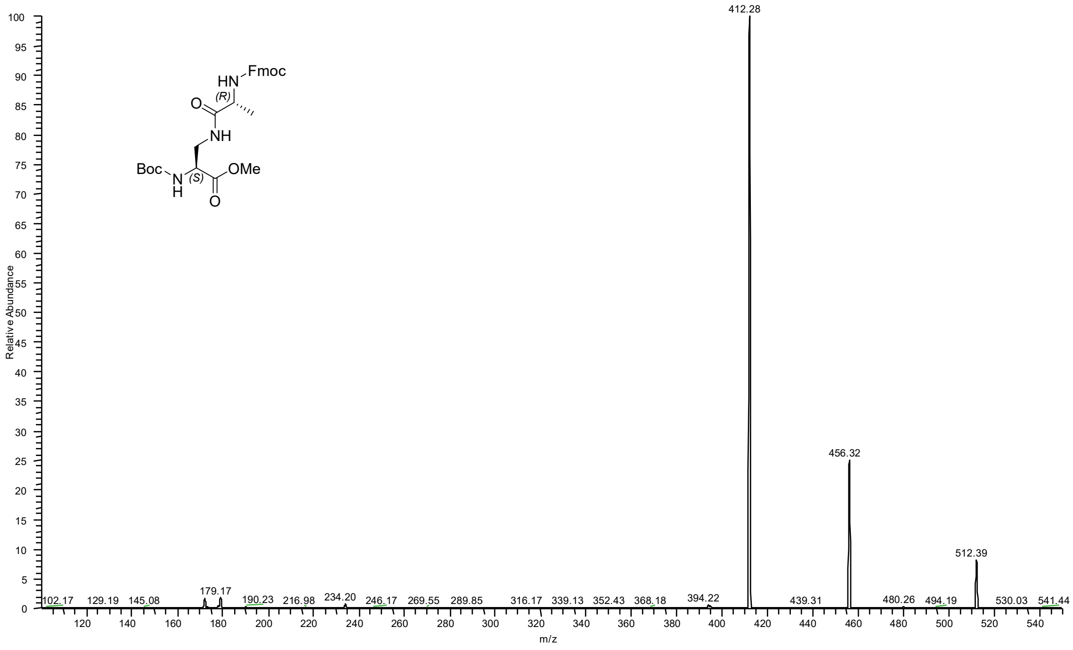

Supplement: Supplementary file 1 [file molecules-25-01313-s001.pdf]
